# The Development of Selective Inhibitors of NagZ: Increased Susceptibility of Gram-Negative Bacteria to β-Lactams

**DOI:** 10.1002/cbic.201300395

**Published:** 2013-09-05

**Authors:** Keith A Stubbs, John-Paul Bacik, G Evan Perley-Robertson, Garrett E Whitworth, Tracey M Gloster, David J Vocadlo, Brian L Mark

**Affiliations:** a School of Chemistry and Biochemistry, University of Western Australia,35 Stirling Highway, Crawley, WA 6009 (Australia); b Department of Microbiology, University of Manitoba,45 Chancellors Circle, Winnipeg, MB R3T2N2 (Canada); c Department of Chemistry, Simon Fraser University, 8888 University Drive,Burnaby, BC V5A1S6 (Canada); d Current address: Bioscience Division, Los Alamos National Laboratory,P. O. Box 1663, Los Alamos, NM 87545 (USA); e Current address: Department of Chemistry, Massachusetts Institute of Technology,77 Massachusetts Avenue, Cambridge, MA 02139 (USA); f Current address: Biomedical Sciences Research Complex, University of St AndrewsNorth Haugh, St Andrews, Fife, KY16 9ST (UK)

**Keywords:** carbohydrates, enzymes, inhibitors, selectivity, synthesis

## Abstract

The increasing incidence of inducible chromosomal AmpC β-lactamases within the clinic is a growing concern because these enzymes deactivate a broad range of even the most recently developed β-lactam antibiotics. As a result, new strategies are needed to block the action of this antibiotic resistance enzyme. Presented here is a strategy to combat the action of inducible AmpC by inhibiting the β-glucosaminidase NagZ, which is an enzyme involved in regulating the induction of AmpC expression. A divergent route facilitating the rapid synthesis of a series of N-acyl analogues of 2-acetamido-2-deoxynojirimycin is reported here. Among these compounds are potent NagZ inhibitors that are selective against functionally related human enzymes. These compounds reduce minimum inhibitory concentration values for β-lactams against a clinically relevant Gram-negative bacterium bearing inducible chromosomal AmpC β-lactamase, *Pseudomonas aeruginosa.* The structure of a NagZ–inhibitor complex provides insight into the molecular basis for inhibition by these compounds.

## Introduction

Resistance to even the most recent generation of β-lactams is steadily developing as various resistance mechanisms disseminate through the bacterial domain. Resistance mechanisms to β-lactams are varied, but the most important involve the β-lactamases—enzymes that deactivate β-lactam antibiotics by cleaving the cyclic amide moiety. One specific class of these enzymes, inducible chromosomal AmpC β-lactamases,^1^–^3^ is increasingly problematic in many Gram-negative bacteria, because these enzymes deactivate a broad range of even the most recent β-lactam antibiotics,^4^–^6^ and are resistant to clinically available β-lactamase inhibitors.^7^, ^8^ As a result, new strategies for blocking the action of this class of enzyme are of considerable interest.^9^

**Scheme 1:**
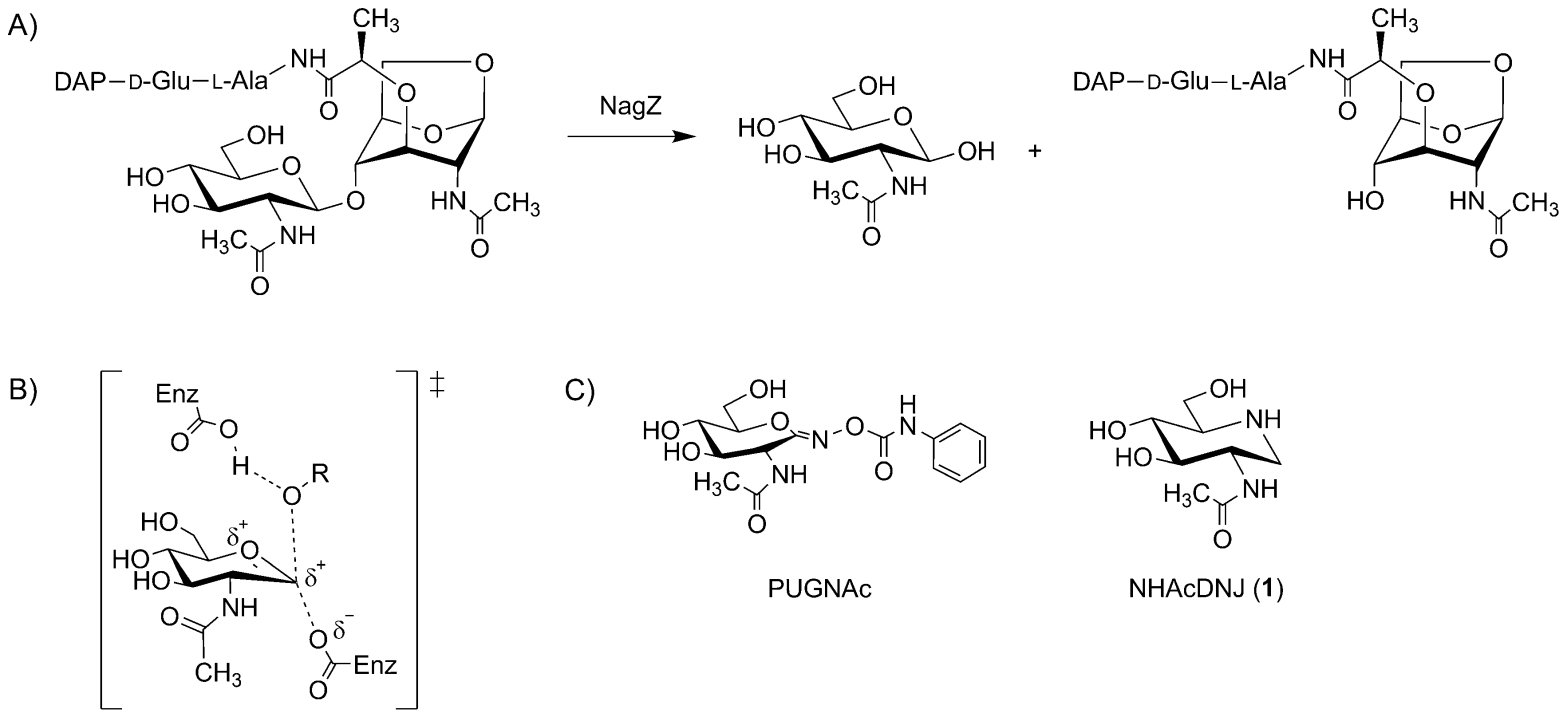
A) NagZ catalyzed hydrolysis of peptidoglycan cell-wall fragments releases the series of 1,6-anhydroMurNAc peptide inducer molecules that activate transcription of *ampC* expression (tripeptide shown). B) The putative transition state of the NagZ catalyzed hydrolysis of *N*-acetylglucosaminides (denoted by ≠). C) Structures of known inhibitors of NagZ enzymes.

AmpC β-lactamase expression depends on the activity of a number of proteins that are engaged in peptidoglycan metabolism. The peptidoglycan is an essential component of the bacterial cell and is a highly cross-linked hetero-polymer that forms an exoskeleton around the organism, defining its shape and protecting it from osmotic lysis.^10^ During normal cell division, a considerable amount of the peptidoglycan is degraded and recycled.^10^,^11^ The resulting GlcNAc-1,6-anhydroMurNAc-peptide degradation products have their non-reducing GlcNAc residue removed by a cytosolic β-glucosaminidase known as NagZ.^12^,^13^ The resulting products are *N*-acetyl-d-glucosamine and a series of 1,6-anhydroMurNAc-tri-, tetra- and pentapeptides (Scheme 1A). These 1,6-anhydroMurNAc catabolic fragments activate the transcription of inducible *ampC* by binding to the transcriptional regulator AmpR.^14^ To prevent the continuous expression of *ampC*, another molecule, UDP-MurNAc-pentapeptide, which is a biosynthetic building block of the cell wall derived from these catabolic products, is involved in repressing *ampC* transcription by binding to AmpR.^14^,^15^ The relative concentrations of these molecules enable bacteria to sense β-lactams and so regulate AmpC expression.^2^

One strategy to combat inducible AmpC would be to inhibit NagZ using small-molecule inhibitors. NagZ, a member of family GH3 (for an overview of the CAZy classification system of glycoside hydrolases see http://www.cazy.org^16^) uses a two-step, double-displacement mechanism involving the formation and breakdown of a covalent glycosyl-enzyme intermediate via oxocarbenium-ion-like transition states (Scheme 1B).^17^–^20^ By inhibiting NagZ, the formation of the inducer molecules comprising 1,6-anhydroMurNAc peptides would be impeded thus leading to reduced AmpC production and increased sensitivity to β-lactams. The approach of targeting NagZ has received recent validation using both chemical,^21^,^22^ and genetic studies,^23^–^25^ while structural studies of NagZ are now enabling the design of improved inhibitors.^26^

The main focus of NagZ inhibitor design has centred around the known β-glucosaminidase inhibitor, *O-*(2-acetamido-2-deoxy-d-glucopyranosylidene)amino *N*-phenylcarbamate (PUGNAc, Scheme 1C).^27^ Despite its potency for NagZ,^21^ a downside to using this molecule in a complex biological context is that it lacks selectivity for NagZ over important human enzymes. PUGNAc has been demonstrated to inhibit family GH84 human O-GlcNAcase (OGA),^28^,^29^ family GH20 human β-hexosaminidases^30^ and family GH89 hexosaminidases related to NAGLU.^31^ An area gaining increasing attention in carbohydrate enzymology is the need for inhibitors with improved selectivity between functionally related enzymes.^32^ Therefore, in an effort to overcome problems associated with concomitant inhibition of these enzymes, a series of PUGNAc derivatives were prepared through modification of the pendant N-acyl chain^21^,^33^ and were found to be selective for NagZ as well as useful at reducing AmpC β-lactamase expression.^21^,^23^

Another molecule that suffers from the same selectivity problem, yet has been used as an inhibitor of β-hexosaminidases, is 2-acetamido-2-deoxynojirimycin (NHAcDNJ, **1**, Scheme 1C). This compound is known to potently inhibit hexosaminidases from families GH20^34^,^35^ and GH89^31^ and has recently been found to inhibit NagZ from *E. coli*.^22^ Based on these observations we envisaged that making modifications to the pendant N-acyl chain of **1** might yield new potent and selective inhibitors for NagZ enzymes. Such compounds might be valuable tools, not only by inhibiting NagZ and thus rendering increased susceptibility to β-lactams of Gram-negative bacteria harbouring inducible *ampC*, but also by aiding improved understanding of the binding of selective inhibitors to this family of enzymes and the role played by NagZ in peptidoglycan recycling.

## Results and Discussion

**Scheme 2:**
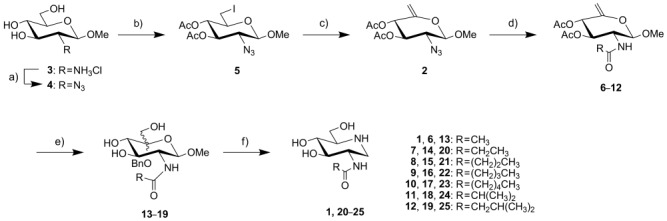
Reagents: a) ImSO_2_N_3_⋅HCl, K_2_CO_3_, CuSO_4_, MeOH; b) i: TsCl, pyridine, CH_2_Cl_2_; ii: NaI, DMF; iii: Ac_2_O, C_5_H_5_N; c) DBU, THF; d) i: PBu_3_, THF, H_2_O; ii: (RCO)_2_O; e) i: 3-chloroperbenzoic acid, CH_2_Cl_2_, BnOH; ii: NaOMe, MeOH; f) NH_4_OAc, Pd(OH)_2_/C, H_2_, MeOH/H_2_O (15:1).

Multiple syntheses of **1** previously described in the literature use *N*-acetyl-d-glucosamine as a starting material.^36^–^40^ To prepare the series of target compounds we envisaged that a divergent synthesis would facilitate their rapid preparation. Accordingly, *N*-acetyl-d-glucosamine was not a viable starting material since we aimed to generate a panel of compounds with various N-acyl groups. We felt, due to the nature of the chemical transformations necessary, that an azido group at C-2 would be stable throughout the synthesis enabling us to prepare a common synthetic intermediate (**2**, Scheme 2), which would be amenable to rapid diversification to generate the desired panel of N-acyl compounds. In addition, the 5,6-alkene could be used in the preparation of the desired iminosugars, through their intermediate ulososides. This general approach has shown value in the preparation of 2-acetamido-1,2-dideoxynojirimycin-lysine hybrids^41^ and other iminosugars.^42^

Starting from the readily accessible hydrochloride **3**, available in three steps from d-glucosamine hydrochloride,^43^ the azido group was introduced to protect the amine moiety using an azido-transfer reagent^44^ to give the triol **4**^45^ (Scheme 2). With triol **4** in hand, a one-pot activation at O-6, using a tosylate group followed by displacement of the tosylate with sodium iodide and in situ acetylation gave the iodide **5** in excellent yield. Elimination of hydroiodic acid across C-5/6 was achieved using 1,8-diazabicyclo[5.4.0]undec-7-ene (DBU) in THF to give the desired intermediate alkene **2**. Treatment of the alkene **2** with tributylphosphine in THF/H_2_O, followed by acylation with the appropriate acyl anhydride gave the series of amides **6**–**12**. Oxidation of the alkenes **6**–**12** with 3-chloroperbenzoic acid provided the presumed intermediate ulososides which were deprotected to give the triols **13**–**19**. Finally, debenzylation by hydrogenolysis and in situ reductive amination of **13**–**19** with ammonium acetate in the presence of hydrogen gratifyingly gave the desired iminosugars **1** and **20**–**25** in excellent overall yield, exclusively as the d-*gluco*-configured materials.

It has been previously established that **1** is a potent competitive inhibitor of lysosomal family GH20 β-hexosaminidases, the lysosomal family GH89 α-hexosaminidase NAGLU, and *E. coli* NagZ. The *K*_i_ value for the human lysosomal family GH20 β-hexosaminidases is 540 nM^35^ (determined against β-hexosaminidase B) and for human NAGLU it is 450 nM.^46^ As previously discussed, developing selective inhibitors of NagZ is important to block NagZ function in bacteria but also to ensure that there is no concomitant inhibition of these human enzymes.

With the synthesised panel of inhibitors in hand, we evaluated them against representative NagZ enzymes found in *Vibrio cholerae* (*Vc*NagZ) and *Salmonella typhimurium* (*St*NagZ) and found them to be potent competitive inhibitors of these enzymes (Table 1). What was also of interest was that increasing the N-acyl chain length leads to a greater increase in *K*_i_ value for both β-hexosaminidase B and NAGLU as compared to NagZ enzymes, consistent with those observed for the *N*-acyl PUGNAc analogues and presumably a consequence of the more spacious active site found in NagZ enzymes.^21^ Furthermore these compounds were also poor inhibitors of OGA, with the parent compound showing a *K*_i_ value of 23 μM, consistent with an independent report for this enzyme.^35^ We find that the selectivity ratio for the NagZ enzymes improves as the chain length increases; this is illustrated best for compound **21**, which shows over 50-fold selectivity for *Vc*NagZ (>20-fold for *St*NagZ) over the human enzymes, whilst retaining potency for the NagZ enzymes. To gain a more detailed understanding of the molecular basis for the inhibition of NagZ enzymes by **21**, which we deemed to be the most potentially promising compound in this series in terms of potency as well as selectivity, we determined the three-dimensional structure of *St*NagZ in complex with **21** at 1.45 Å resolution (FigureFigure 1).

**Table 1:** Inhibition constants and selectivity ratio (S.R.) of inhibitors for O-GlcNAcase, β-hexosaminidase B, NAGLU, *Vc*NagZ and *St*NagZ.

Compound	O-GlcNAcase *K*_i_	β-Hexosaminidase B	NAGLU *K*_i_	*Vc*NagZ *K*_i_	*St*NagZ *K*_i_	S.R.	S.R.
	[μM]	*K*_i_ [μM]	[μM]	[μM]	[μM]	(*K*_i_ OGA/*K*_i_ *Vc*NagZ)	(*K*_i_ OGA/*K*_i_ *St*NagZ)
**1**	23	0.54^35^	0.45^46^	8.5	25.2	2.8	0.94
**20**	130	61	96	4.2	45.4	31	2.9
**21**	>500	1460	>1000	9.4	23.2	>53	>22
**22**	>1000	>5000	>1000	110	47.3	>9	>21
**23**	>1000	>5000	>1000	1135	102	>0.9	>10
**24**	>1000	670	800	15.8	24.6	63	>40
**25**	>1000	>5000	>1000	555	294	>1.8	>3.4

**Figure 1:**
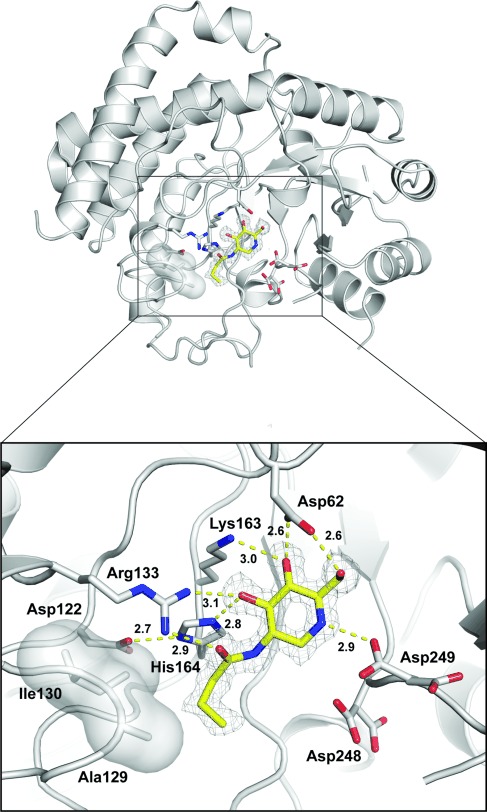
Crystal structure of *St*NagZ bound to 21. Active-site residues and **21** are drawn as sticks with oxygen and nitrogen atoms shown in red and blue, respectively. Carbon atoms of the enzyme are shown in grey, while the carbon atoms of the inhibitor are yellow. The hydrophobic surface formed by side chains of Ala129 and Ile130, against which the butyl chain sits, is shown in grey. The catalytic nucleophile, Asp248, is shown in stick format. Occupancy values for the dual conformations of Asp248 are 0.48/0.52, and 0.53/0.47 for Asp249 (flipped in or out towards the inhibitor, respectively). Hydrogen bonds are shown as yellow dashed lines. Electron density around **21** is a maximum-likelihood-weighted omit map (*F*_obs_−*F*_calcd_) contoured to 2.5*σ*.

With the enzyme–inhibitor complex we find that the pseudo-glycoside ring structure of **21** adopts a relaxed ^4^*C*_1_ chair conformation that closely resembles that observed for the reaction product GlcNAc. Accordingly, similar hydrogen-bonding interactions are observed between **21** and residues in the active site (for a detailed description of the structure and mechanism of NagZ, see ref. 20). However, we also note that the NH group within the ring of **21**, which replaces the endocyclic oxygen of GlcNAc, forms a hydrogen bond with the side-chain of Asp249, which likely confers the increased binding affinity relative to GlcNAc. The butyl moiety of the N-acyl group of **21** appears to form hydrophobic interactions with a small surface created by the side chains of Ala129 and Ile130 adopting a similar conformation as that seen in the *Vc*NagZ structures bound to PUGNAc-derived inhibitors.^21^,^26^ Finally, the structure shows the butyl chain is exposed to solvent on the outer side, supporting the idea that the relatively open active-site arrangement of NagZ accounts for the much greater selectivity demonstrated toward the bacterial hydrolases by the NHAcDNJ*-*derived inhibitors with more bulky modified N-acyl chains.

We next set out to obtain insight into whether these compounds could increase the susceptibility of bacteria harbouring inducible AmpC β-lactamase to β-lactams. We therefore evaluated them against *Pseudomonas aeruginosa* PA01, a Gramnegative bacterium that harbours a chromosomally inducible AmpC β-lactamase.^47^ This opportunistic pathogen is problematic for patients suffering from cystic fibrosis, severe burns and pulmonary disease.^48^–^50^ Importantly, *P. aeruginosa* PA01 contains a functional NagZ and strains lacking the *nagZ* gene are known to have increased susceptibility to β-lactams, supporting the validity of this strain in such β-lactam susceptibility assays.^23^,^24^

A series of β-lactam antibiotics, cefoxitin, ceftazidime, ampicillin, the monobactam aztreonam and the carbapenem imipenem, were chosen as they are commonly used in clinical antibiotic susceptibility experiments. Using minimal inhibitory concentration (MIC) assays we found that cultures treated with the selective inhibitor **21** are more susceptible to these β-lactam antibiotics when compared to control cultures that were not treated with **21** (Table 2).

**Table 2:** Susceptibility of *P. aeruginosa* PA01 against various β-lactam antibiotics.

Antibiotic	MIC [μgmL^−1^][a]		Clearing radius [mm][b]	
	−21	+21	−21	+21
Ceftazidime	2	0.5	12.2	14.3
Aztreonam	1	0.5	13.8	16.0
Imipenem	8	4	11.9	13.3
Ampicillin	512	128		
Cefoxitin	2048	512		

[a] MIC determined by standard serial dilution.

[b] Susceptibility determined in an agar diffusion assay using 6 mm filter disks loaded with 30 μg of antibiotic with or without **21**. The zone of clearance was measured after incubation overnight.

To use a separate assessment of antibiotic susceptibility we took the more potent β-lactams, ceftazidime, aztreonam and imipenem and assessed them in agar diffusion assays (Table 2). In accord with the MIC data we found that agar-diffusion assays gave similar results, revealing enhanced susceptibility to β-lactams in the presence of **21**. As a control we evaluated **21** as an inhibitor of bacterial growth but observed no differences in growth rates even at a concentration of 1 mM (data not shown), indicating that the inhibitor, on its own, is not antibacterial or bacteriostatic. Of note is that the concentration of **21** required to give rise to the increased susceptibility observed is fivefold less than the concentration of the PUGNAc analogue *O*-(2-deoxy-2-*N*-2-ethylbutyryl-d-glucopyranosylidene)amino *N*-phenylcarbamate (EtBuPUG) used previously to induce a similar effect.^23^ Together, these results suggest that **21** might gain more ready access to the cytosol, either through a transporter protein or passive diffusion as compared to EtBuPUG.

## Conclusions

The incidence of inducible chromosomal AmpC β-lactamases is increasing and since these enzymes deactivate a broad range of β-lactam antibiotics they are a pressing health issue. We have devised a divergent route that enables the rapid synthesis of a series of potent and selective NHAcDNJ-based inhibitors bearing different N*-*acyl groups that target NagZ, an important enzyme in the regulation of AmpC activity. One of these compounds reduces MIC values for β-lactams against a clinically relevant Gram-negative bacterium bearing inducible chromosomal AmpC β-lactamase, *P. aeruginosa*. Using a structure of a NagZ–inhibitor complex we provide insight into the molecular basis for the selectivity and potency of this inhibitor. It is anticipated that further structure-guided refinement will lead to candidates with increased potency for NagZ and, using the strategy outlined here, systematic elaboration of other β-*N*-acetylglucosaminidase inhibitor scaffolds might also yield selective and potent inhibitors for NagZ.

## Experimental Section

**General methods for chemical synthesis:** ^1^H and ^13^C NMR spectra were recorded on a Bruker ARX500 (500 MHz for ^1^H and 125 MHz for ^13^C) or a Bruker AV600 (600 MHz for ^1^H and 150 MHz for ^13^C; chemical shifts quoted relative to CHCl_3_ for CDCl_3_ and CH_3_OH for D_2_O where appropriate) spectrometer. Mass spectra were recorded with a VG-Autospec spectrometer using the fast atom bombardment technique, with 3-nitrobenzyl alcohol as a matrix. Elemental analyses of all synthesised compounds used in enzyme assays were performed at the Australian National University Microanalytical Facility. Flash chromatography was performed on silica gel (BDH) with the specified solvents. Thin-layer chromatography (TLC) was effected on Merck silica gel 60 F_254_ aluminium-backed plates that were stained by heating (>200°C) with 5% sulfuric acid in EtOH. Percentage yields for chemical reactions as described are quoted only for those compounds that were purified by recrystallization or by column chromatography, and purity was assessed using TLC or ^1^H NMR spectroscopy.

**Methyl 3,4-di-*O*-acetyl-2-azido-2-deoxy-6-iodo-β-d-glucopyranoside (5):**­ *p*-Toluenesulfonyl chloride (3.9 g, 20 mmol) was added to a solution of azide **4**^45^ (4.0 g, 18 mmol) in pyridine (20 mL) and CH_2_Cl_2_ (80 mL) and the solution was stirred (RT, 6 h). The reaction was quenched by the addition of water and the resultant mixture stirred (1 h). The organic layer was then collected and was washed with water (1×50 mL), aq. HCl (1M, 1×50 mL), saturated aq. NaHCO_3_ solution (1×50 mL), brine (1×20 mL), dried (MgSO_4_), filtered and concentrated. The residue was then dissolved in DMF (60 mL) and to it was added NaI (8.0 g, 53 mmol) and the resultant mixture stirred (100°C, 4 h). The mixture was then concentrated and the resultant residue diluted with EtOAc (150 mL) and was washed with water (2×20 mL), brine (1×20 mL), dried (MgSO_4_), filtered and concentrated. The residue was then dissolved in a mixture of CH_2_Cl_2_ (30 mL) and pyridine (10 mL) and Ac_2_O (10 mL) was added. The resultant solution was left at RT overnight. The mixture was quenched with MeOH and concentrated. The resultant residue was then diluted with CH_2_Cl_2_ (100 mL) and washed with water (2×20 mL), aq. HCl (1M, 1×50 mL), saturated aq. NaHCO_3_ solution (1×50 mL), brine (1×20 mL), dried (MgSO_4_), filtered and concentrated. Flash chromatography of the resultant residue (EtOAc/hexane 3:7) yielded **5** as a colourless oil (4.8 g, 64%, over three steps). *R*_f_=0.7 (EtOAc/hexane 2:3); ^1^H NMR (600 MHz, CDCl_3_): *δ*=4.98 (dd, *J*=9.3, 9.3 Hz, 1H), 4.80 (dd, *J*=9.5, 9.5 Hz, 1H), 4.31 (d, *J*=8.0 Hz, 1H), 3.63 (s, 3H), 3.49–3.44 (m, 2H), 3.29 (dd, *J*=2.7, 11.0 Hz, 1H), 3.14 (dd, *J*=8.4, 11.0 Hz, 1H), 2.07 (s, 3H), 2.04 ppm (s, 3H); ^13^C NMR (150 MHz, CDCl_3_): *δ*=169.9, 169.6, 102.5, 73.2, 72.3, 72.2, 63.8, 57.4, 20.64, 20.63, 2.8 ppm; *ν*_max_=2114 cm^−1^ (N_3_); HRMS: *m*/*z* calcd: 414.0162 [*M*+H]^+^, found: 414.0156; elemental analysis calcd (%) for C_11_H_16_IN_3_O_6_: C 31.98, H 3.90, N 10.17; found: C 31.93, H 3.92, N 10.15.

**Methyl 3,4-di-*O*-acetyl-2-azido-2,6-dideoxy-β-d-*xylo*-hex-5-enoside (2):** DBU (4.9 mL, 33 mmol) was added to iodide **5** (4.5 g, 11 mmol) in THF (40 mL) and the mixture refluxed (2 h). The mixture was then concentrated and the resultant residue diluted with EtOAc (150 mL) and was washed with water (2×40 mL), ice-cold aq. HCl (1M, 1×50 mL), saturated aq. NaHCO_3_ solution (1×50 mL), brine (1×50 mL), dried (MgSO_4_), filtered and concentrated. Flash chromatography of the resultant residue (EtOAc/hexane 3:7) yielded **2** as a colourless oil (2.5 g, 80%). *R*_f_=0.4 (EtOAc/hexane 1:4); ^1^H NMR (500 MHz, CDCl_3_): *δ*=5.41 (ddd, *J*=1.6, 1.6, 10.0 Hz, 1H), 4.93 (dd, *J*=9.2, 9.2 Hz, 1H), 4.82 (dd, *J*=1.6, 1.6 Hz, 1H), 4.59 (dd, *J*=1.6, 1.6 Hz, 1H), 4.37 (d, *J*=7.2 Hz, 1H), 3.59 (s, 3H), 2.08 (s, 3H), 2.07 ppm (s, 3H); ^13^C NMR (125 MHz, CDCl_3_): *δ*=169.6, 169.3, 103.2, 97.5, 71.4, 69.2, 63.4, 57.3, 20.6 ppm; *ν*_max_=2108 cm^−1^ (N_3_); HRMS: *m*/*z* calcd: 286.1039 [*M*+H]^+^, found: 286.1028; elemental analysis calcd (%) for C_11_H_15_N_3_O_6_: C 46.32, H 5.30, N 14.73; found: C 46.28, H 5.33, N 14.80.

**General procedure for formation of methyl 3,4-di-*O*-acetyl-2-acylamido-2,6-dideoxy-β-d-*xylo*-hex-5-enosides (6**–**12):** Tributyl phosphine (0.2 mL, 0.8 mmol) was added to azide **2** (200 mg, 0.7 mmol) in a solution of THF (5 mL) and H_2_O (0.5 mL) at 0°C and the solution stirred. This was followed by the addition of the appropriate acyl anhydride (3 equiv) and the mixture stirred (5 h). Concentration followed by flash chromatography of the residue (EtOAc/hexane 7:3) gave the desired compounds **6**–**12** in yields ranging from 52% to 73%.

**Methyl 2-acetamido-3,4-di-*O*-acetyl-2,6-dideoxy-β-d-*xylo*-hex-5-enoside (6):** Yield: 60%. ^1^H and ^13^C NMR spectra were consistent with those found in the literature.^41^

**Methyl 3,4-di-*O*-acetyl-2,6-dideoxy-2-propamido-β-d-*xylo*-hex-5-enoside (7):** Yield: 73%; *R*_f_=0.3 (EtOAc/hexane 7:3); ^1^H NMR (600 MHz, CDCl_3_): *δ*=5.81 (d, *J*=8.8 Hz, 1H), 5.61 (ddd, *J*=1.3, 1.3, 7.5 Hz, 1H), 4.94 (dd, *J*=6.0, 7.5 Hz, 1H), 4.78 (dd, *J*=1.3, 1.3 Hz, 1H), 4.66 (d, *J*=4.0 Hz, 1H), 4.57 (dd, *J*=1.3, 1.3 Hz, 1H), 4.24 (ddd, *J*=4.0, 6.0, 8.8 Hz, 1H), 3.48 (s, 3H), 2.24–2.19 (m, 2H), 2.11 (s, 3H), 2.06 (s, 3H), 1.14 ppm (t, *J*=7.5 Hz, 3H); ^13^C NMR (150 MHz, CDCl_3_): *δ*=173.3, 170.4, 168.9, 150.5, 102.3, 97.3, 71.4, 68.6, 56.2, 52.1, 29.6, 20.8, 20.7, 9.5 ppm; HRMS: *m*/*z* calcd: 316.1396 [*M*+H]^+^, found: 316.1371; elemental analysis calcd (%) for C_14_H_21_NO_7_: C 53.33, H 6.71, N 4.44; found: C 53.30, H 6.66, N 4.51.

**Methyl 3,4-di-*O*-acetyl-2-butamido-2,6-dideoxy-β-d-*xylo*-hex-5-enoside (8):** Yield: 70%; *R*_f_=0.4 (EtOAc/hexane 7:3); ^1^H NMR (600 MHz, CDCl_3_): *δ*=5.76 (d, *J*=9.3 Hz, 1H), 5.61 (d, *J*=7.5 Hz, 1H), 4.95 (dd, *J*=6.3, 6.4 Hz, 1H), 4.79 (s, 1H), 4.66 (d, *J*=4.1 Hz, 1H), 4.57 (s, 1H), 4.24 (ddd, *J*=4.1, 7.5, 9.3 Hz, 1H), 3.48 (s, 3H), 2.16 (t, *J*=8.0 Hz, 2H), 2.12 (s, 3H), 2.06 (s, 3H), 1.68–1.63 (m, 2H), 0.94 ppm (t, *J*=7.3 Hz, 3H); ^13^C NMR (150 MHz, CDCl_3_): *δ*=172.5, 170.4, 169.0, 150.6, 102.4, 97.2, 71.4, 68.6, 56.2, 52.2, 38.6, 20.84, 20.8, 18.9, 13.6 ppm; HRMS: *m*/*z* calcd: 330.1553 [*M*+H]^+^, found: 330.1564; elemental analysis calcd (%) for C_15_H_23_NO_7_: C 54.70, H 7.04, N 4.25; found: C 54.65, H 7.07, N 4.33.

**Methyl 3,4-di-*O*-acetyl-2,6-dideoxy-2-valeramido-β-d-*xylo*-hex-5-enoside (9):** Yield: 67%; *R*_f_=0.5 (EtOAc/hexane 7:3); ^1^H NMR (600 MHz, CDCl_3_): *δ*=5.79 (d, *J*=8.6 Hz, 1H), 5.60 (d, *J*=7.5 Hz, 1H), 4.95 (dd, *J*=6.4, 7.3 Hz, 1H), 4.78 (s, 1H), 4.65 (d, *J*=4.1 Hz, 1H), 4.57 (s, 1H), 4.23 (ddd, *J*=4.1, 6.4, 8.6 Hz, 1H), 3.48 (s, 3H), 2.19–2.16 (m, 2H), 2.12 (s, 3H), 2.05 (s, 3H), 1.61–1.56 (m, 2H), 1.36–1.29 (m, 2H), 0.90 ppm (t, *J*=6.8 Hz, 3H); ^13^C NMR (150 MHz, CDCl_3_): *δ*=172.7, 170.4, 169.0, 150.6, 102.4, 97.2, 71.4, 68.6, 56.2, 52.2, 36.7, 27.5, 22.2, 20.82, 20.8, 13.7 ppm; HRMS: *m*/*z* calcd: 344.1709 [*M*+H]^+^, found: 344.1722; elemental analysis calcd (%) for C_16_H_25_NO_7_: C 55.97, H 7.34, N 4.08; found: C 55.89, H 7.39, N 4.09.

**Methyl 3,4-di-*O*-acetyl-2,6-dideoxy-2-hexamido-β-d-*xylo*-hex-5-enoside (10):** Yield: 52%; *R*_f_=0.7 (EtOAc/hexane 7:3); ^1^H NMR (500 MHz, CDCl_3_): *δ*=5.76 (d, *J*=8.7 Hz, 1H), 5.60 (dt, *J*=1.1, 1.1, 7.6 Hz, 1H), 4.95 (dd, *J*=6.3, 7.4 Hz, 1H), 4.78 (d, *J*=1.1 Hz, 1H), 4.65 (d, *J*=4.1 Hz, 1H), 4.57 (d, *J*=1.1 Hz, 1H), 4.24 (ddd, *J*=4.1, 6.3, 8.7 Hz, 1H), 3.49 (s, 3H), 2.19–2.16 (m, 2H), 2.12 (s, 3H), 2.06 (s, 3H), 1.65–1.59 (m, 4H), 1.35–1.25 (m, 4H), 0.89 ppm (t, *J*=6.8 Hz, 3H); ^13^C NMR (125 MHz, CDCl_3_): *δ*=172.7, 170.4, 169.0, 150.6, 102.4, 97.2, 71.4, 68.6, 56.2, 52.2, 36.6, 31.3, 25.2, 22.3, 20.84, 20.8, 13.9 ppm; HRMS: *m*/*z* calcd: 358.1866 [*M*+H]^+^, found: 358.1857; elemental analysis calcd (%) for C_17_H_27_NO_7_: C 57.13, H 7.61, N 3.92; found: C 57.01, H 7.53, N 3.99.

**Methyl 3,4-di-*O*-acetyl-2,6-dideoxy-2-isobutamido-β-d-*xylo*-hex-5-enoside (11):** Yield: 54%; *R*_f_=0.45 (EtOAc/hexane 7:3); ^1^H NMR (500 MHz, CDCl_3_): *δ*=5.85 (d, *J*=8.4 Hz, 1H), 5.60 (d, *J*=7.5 Hz, 1H), 4.95 (dd, *J*=6.3, 6.3 Hz, 1H), 4.79 (s, 1H), 4.65 (d, *J*=4.0 Hz, 1H), 4.57 (s, 1H), 4.22 (ddd, *J*=4.0, 6.3, 8.4 Hz, 1H), 3.48 (s, 3H), 2.38–2.33 (m, 1H), 2.12 (s, 3H), 2.05 (s, 3H), 1.15 (d, *J*=6.9 Hz, 3H), 1.14 ppm (d, *J*=6.9 Hz, 3H); ^13^C NMR (125 MHz, CDCl_3_): *δ*=176.5, 170.4, 168.9, 150.5, 102.4, 97.5, 71.3, 68.6, 56.2, 51.9, 35.5, 20.83, 20.8, 19.4, 19.3 ppm; HRMS: *m*/*z* calcd: 330.1553 [*M*+H]^+^, found: 330.1570; elemental analysis calcd (%) for C_15_H_23_NO_7_: C 54.70, H 7.04, N 4.25; found: C 54.63, H 7.09, N 4.28.

**Methyl 3,4-di-*O*-acetyl-2,6-dideoxy-2-isovalermido-β-d-*xylo*-hex-5-enoside (12):** Yield: 68%; *R*_f_=0.5 (EtOAc/hexane 7:3); ^1^H NMR (600 MHz, CDCl_3_): *δ*=5.75 (d, *J*=8.9 Hz, 1H), 5.60 (dt, *J*=1.3, 1.3, 7.6 Hz, 1H), 4.96 (dd, *J*=6.5, 7.5 Hz, 1H), 4.79 (d, *J*=1.3 Hz, 1H), 4.64 (d, *J*=4.2 Hz, 1H), 4.57 (d, *J*=1.3 Hz, 1H), 4.24 (ddd, *J*=4.2, 6.5, 8.9 Hz, 1H), 3.48 (s, 3H), 2.12 (s, 3H), 2.10–2.04 (m, 3H), 2.06 (s, 3H), 0.96–0.93 ppm (m, 6H); ^13^C NMR (150 MHz, CDCl_3_): *δ*=172.1, 170.4, 169.0, 150.6, 102.5, 97.1, 71.4, 68.7, 56.2, 52.2, 46.0, 26.1, 22.35, 22.3, 20.82, 20.8 ppm; HRMS: *m*/*z* calcd: 344.1709 [*M*+H]^+^, found: 344.1729; elemental analysis calcd (%) for C_16_H_25_NO_7_: C 55.97, H 7.34, N 4.08; found: C 56.02, H 7.41, N 4.04.

**General procedure for formation of methyl (*5R*/*S*)-2-acylamino-5-*C*-benzyloxy-2-deoxy-β-d-*xylo*-hexopyranosides (13**–**19):** To a 1% solution of **6**–**12** (0.5 mmol) in a mixture of CH_2_Cl_2_/benzyl alcohol (1:1 *v*/*v*, 12 mL), 3-chloroperbenzoic acid (70%, 0.6 mmol) was added, and the mixture was stirred (2 h). The mixture was then diluted with CH_2_Cl_2_ (20 mL) and washed with saturated aq. NaHCO_3_ solution (1×50 mL), dried (MgSO_4_), filtered and concentrated. The resulting residue was chromatographed (EtOAc) to yield a residue that was dissolved in MeOH and treated with sodium methoxide (10 mg) and the solution stirred (RT, 30 min). The mixture was quenched with resin (Amberlite IR-120, H^+^), filtered and concentrated to give **13**–**19** as a mixture of diastereomers at C-5 from which the major (5*S*) epimer was purified by flash chromatography (MeOH/CHCl_3_ 1:9) in yields ranging from 53% to 68% over two steps.

**Methyl (*5R*/*S*)-2-acetamido-5-*C*-benzyloxy-2-deoxy-β-d-*xylo*-hexopyranoside (13):** Yield: 68%. ^1^H and ^13^C NMR spectra of the (5*S*) epimer were consistent with those found in the literature.^41^

**Methyl (*5R*/*S*)-5-*C*-benzyloxy-2-deoxy-2-propamido-β-d-*xylo*-hexopyranoside (14):** Yield: 64%. (5*S*) epimer—*R*_f_=0.2 (EtOAc); ^1^H NMR (500 MHz, CD_3_OD): *δ*=7.45–7.42 (m, 2H), 7.32–7.28 (m, 2H), 7.25–7.23 (m, 1H), 4.76 (ABq, *J*=10.9 Hz, 1H), 4.71–4.68 (m, 2H), 4.02 (dd, *J*=7.0, 9.7 Hz, 1H), 3.89–3.83 (m, 3H), 3.71 (dd, *J*=7.9, 9.6 Hz, 1H), 3.44 (s, 3H), 2.26–2.21 (m, 2H), 1.12 ppm (t, *J*=7.6 Hz, 3H); ^13^C NMR (125 MHz, CD_3_OD): *δ*=177.4, 140.0, 129.2, 129.0, 128.3, 102.9, 101.9, 76.0, 73.6, 64.6, 62.3, 56.8, 56.3, 30.4, 10.3 ppm; HRMS: *m*/*z* calcd: 356.1709 [*M*+H]^+^, found: 356.1711; elemental analysis calcd (%) for C_17_H_25_NO_7_: C 57.45, H 7.09, N 3.94; found: C 57.28, H 7.01, N 3.99.

**Methyl (*5R*/*S*)-5-*C*-benzyloxy-2-butamido-2-deoxy-β-d-*xylo*-hexopyranoside (15):** Yield: 53%. (5*S*) epimer—*R*_f_=0.25 (EtOAc); ^1^H NMR (500 MHz, CD_3_OD): *δ*=7.45–7.41 (m, 2H), 7.32–7.28 (m, 2H), 7.25–7.22 (m, 1H), 4.75 (ABq, *J*=9.0 Hz, 1H), 4.71–4.68 (m, 2H), 4.03 (dd, *J*=7.4, 9.6 Hz, 1H), 3.90–3.83 (m, 3H), 3.71 (dd, *J*=8.1, 9.3 Hz, 1H), 3.44 (s, 3H), 2.20 (t, *J*=6.4 Hz, 2H), 1.69–1.62 (m, 2H), 0.96 ppm (t, *J*=7.4 Hz, 3H); ^13^C NMR (125 MHz, CD_3_OD): *δ*=176.5, 140.0, 129.1, 129.0, 128.3, 102.8, 101.9, 76.1, 73.5, 64.6, 62.3, 56.8, 56.3, 39.3, 20.3, 13.9 ppm; HRMS: *m*/*z* calcd: 370.1866 [*M*+H]^+^, found: 370.1889; elemental analysis calcd (%) for C_18_H_27_NO_7_: C 58.52, H 7.37, N 3.79; found: C 58.49, H 7.51, N 3.71.

**Methyl (*5R*/*S*)-5-*C*-benzyloxy-2-deoxy-2-valeramido-β-d-*xylo*-hexopyranoside (16):** Yield: 68%. (5*S*) epimer—*R*_f_=0.3 (EtOAc); ^1^H NMR (600 MHz, CD_3_OD): *δ*=7.45–7.43 (m, 2H), 7.32–7.28 (m, 2H), 7.25–7.22 (m, 1H), 4.76 (ABq, *J*=10.9 Hz, 1H), 4.71–4.68 (m, 2H), 4.02 (dd, *J*=7.3, 9.6 Hz, 1H), 3.89–3.82 (m, 3H), 3.71 (dd, *J*=8.1, 9.3 Hz, 1H), 3.44 (s, 3H), 2.24–2.20 (m, 2H), 1.62–1.58 (m, 2H), 1.40–1.35 (m, 2H), 0.94 ppm (t, *J*=7.3 Hz, 3H); ^13^C NMR (150 MHz, CD_3_OD): *δ*=176.7, 140.0, 129.2, 129.0, 128.3, 102.9, 101.9, 76.1, 73.6, 64.6, 62.3, 56.8, 56.3, 37.2, 29.1, 23.2, 14.1 ppm; HRMS: *m*/*z* calcd: 384.2022 [*M*+H]^+^, found: 384.2010; elemental analysis calcd (%) for C_19_H_29_NO_7_: C 59.52, H 7.62, N 3.65; found: C 59.64, H 7.59, N 3.61.

**Methyl (*5R*/*S*)-5-*C*-benzyloxy-2-deoxy-2-hexamido-β-d-*xylo*-hexopyranoside (17):** Yield: 61%. (5*S*) epimer—*R*_f_=0.4 (EtOAc); ^1^H NMR (500 MHz, CD_3_OD): *δ*=7.45–7.41 (m, 2H), 7.32–7.28 (m, 2H), 7.25–7.21 (m, 1H), 4.76–4.70 (m, 3H), 4.03 (dd, *J*=7.2, 9.5 Hz, 1H), 3.90–3.84 (m, 3H), 3.73 (dd, *J*=8.1, 9.6 Hz, 1H), 3.44 (s, 3H), 2.24–2.20 (m, 2H), 1.66–1.59 (m, 2H), 1.40–1.30 (m, 4H), 0.91 ppm (t, *J*=6.8 Hz, 3H); ^13^C NMR (125 MHz, CD_3_OD): *δ*=176.7, 140.0, 129.1, 129.0, 128.3, 102.8, 101.8, 76.0, 73.4, 64.5, 62.3, 56.8, 56.3, 37.3, 32.3, 26.6, 23.4, 14.3 ppm; HRMS: *m*/*z* calcd: 398.2179 [*M*+H]^+^, found: 398.2171; elemental analysis calcd (%) for C_20_H_31_NO_7_: C 60.44, H 7.86, N 3.52; found: C 60.51, H 7.93, N 3.51.

**Methyl (*5R*/*S*)-5-*C*-benzyloxy-2-deoxy-2-isobutamido-β-d-*xylo*-hexopyranoside (18):** Yield: 55%. (5*S*) epimer—*R*_f_=0.3 (EtOAc); ^1^H NMR (500 MHz, CD_3_OD): *δ*=7.45–7.42 (m, 2H), 7.32–7.28 (m, 2H), 7.25–7.21 (m, 1H), 4.76 (ABq, *J*=11.0 Hz, 1H), 4.71–4.68 (m, 2H), 4.00 (dd, *J*=6.9, 9.3 Hz, 1H), 3.90–3.82 (m, 3H), 3.73 (dd, *J*=7.8, 9.3 Hz, 1H), 3.44 (s, 3H), 2.48–2.42 (m, 1H), 1.14 (d, *J*=6.7 Hz, 3H), 1.13 ppm (d, *J*=6.7 Hz, 3H); ^13^C NMR (125 MHz, CD_3_OD): *δ*=180.5, 140.0, 129.2, 129.0, 128.3, 102.9, 102.0, 76.0, 73.5, 64.6, 62.2, 56.8, 56.2, 36.5, 20.0, 19.8 ppm; HRMS: *m*/*z* calcd: 370.1866 [*M*+H]^+^, found: 370.1872; elemental analysis calcd (%) for C_18_H_27_NO_7_: C 58.52, H 7.37, N 3.79; found: C 58.50, H 7.31, N 3.75.

**Methyl (*5R*/*S*)-5-*C*-benzyloxy-2-deoxy-2-isovaleramido-β-d-*xylo*-hexopyranoside (19):** Yield: 64%. (5*S*) epimer—*R*_f_=0.4 (EtOAc); ^1^H NMR (600 MHz, CD_3_OD): *δ*=7.45–7.42 (m, 2H), 7.33–7.29 (m, 2H), 7.25–7.21 (m, 1H), 4.75 (ABq, *J*=11.0 Hz, 1H), 4.72–4.69 (m, 2H), 4.04 (dd, *J*=7.4, 9.7 Hz, 1H), 3.90–3.83 (m, 3H), 3.72 (dd, *J*=8.1, 9.5 Hz, 1H), 3.44 (s, 3H), 2.12–2.05 (m, 3H), 0.98–0.94 ppm (m, 6H); ^13^C NMR (150 MHz, CD_3_OD): *δ*=176.0, 140.0, 129.1, 129.0, 128.3, 102.8, 101.8, 76.1, 73.5, 64.6, 62.3, 56.7, 56.2, 46.7, 27.5, 22.8, 22.6 ppm; HRMS: *m*/*z* calcd: 384.2022 [*M*+H]^+^, found: 384.2024, elemental analysis calcd (%) for C_19_H_29_NO_7_: C 59.52, H 7.62, N 3.65; found: C 59.60, H 7.65, N 3.70.

**General procedure for formation of 2-acylamido-1,5-imino-1,2,5-trideoxy-d-glucitols (1, 20**–**25):** To a solution of **13**–**19** in MeOH/H_2_O (15:1 *v*/*v*, 0.03M), NH_4_HCO_2_ (1 equiv) and 20% Pd(OH)_2_/C (0.1 equiv) were added, and the heterogeneous reaction mixture was stirred under hydrogen at RT and ambient pressure (1 atm, 48 h). After filtration and evaporation, the resulting residue was chromatographed (CHCl_3_/MeOH/conc. NH_3_ 12:8:1) to yield the desired compounds **1**, **20**–**25** in yields ranging from 48 to 63%.

**2-Acetamido-1,5-imino-1,2,5-trideoxy-d-glucitol (1):** Yield: 58%. ^1^H and ^13^C NMR spectra were consistent with those found in the literature.^39^

**1,5-Imino-2-propamido-1,2,5-trideoxy-d-glucitol (20):** Yield: 52%; *R*_f_=0.25 (CHCl_3_/MeOH/conc. NH_3_ 12:8:1); ^1^H NMR (600 MHz, CD_3_OD): *δ*=3.86–3.80 (m, 2H), 3.71 (dd, *J*=5.9, 11.4 Hz, 1H), 3.38–3.34 (m, 2H), 3.21 (dd, *J*=4.7, 12.5 Hz, 1H), 2.71–2.66 (m, 1H), 2.56 (dd, *J*=11.9, 11.9 Hz, 1H), 2.26–2.20 (m, 2H), 1.12 ppm (t, *J*=7.6 Hz, 3H); ^13^C NMR (150 MHz, CD_3_OD): *δ*=177.5, 76.7, 72.6, 62.5, 61.4, 52.3, 47.8, 30.1, 10.3 ppm; HRMS: *m*/*z* calcd: 219.1345 [*M*+H]^+^, found: 219.1344; elemental analysis calcd (%) for C_9_H_18_N_2_O_4_: C 49.53, H 8.31, N 12.84; found: C 49.51, H 8.26, N 12.89.

**2-Butamido-1,5-imino-1,2,5-trideoxy-d-glucitol (21):** Yield: 63%; *R*_f_=0.4 (CHCl_3_/MeOH/conc. NH_3_ 12:8:1); ^1^H NMR (500 MHz, CD_3_OD): *δ*=3.90–3.85 (m, 2H), 3.75 (dd, *J*=5.3, 11.5 Hz, 1H), 3.48 (dd, *J*=9.7, 9.7 Hz, 1H), 3.43 (dd, *J*=9.4, 9.4 Hz, 1H), 3.21 (dd, *J*=4.8, 12.7 Hz, 1H), 2.81–2.76 (m, 1H), 2.64 (dd, *J*=11.9, 11.9 Hz, 1H), 2.28–2.20 (m, 2H), 1.65–1.59 (m, 2H), 0.91 ppm (t, *J*=7.5 Hz, 3H); ^13^C NMR (125 MHz, CD_3_OD): *δ*=178.4, 75.9, 71.7, 61.2, 60.9, 51.6, 47.0, 38.7, 19.9, 13.6 ppm; HRMS: *m*/*z* calcd: 233.1501 [*M*+H]^+^, found: 233.1516; elemental analysis calcd (%) for C_10_H_20_N_2_O_4_: C 51.71, H 8.68, N 12.06; found: C 51.77, H 8.59, N 12.13.

**1,5-Imino-1,2,5-trideoxy-2-valeramido-d-glucitol (22):** Yield: 61%; *R*_f_=0.5 (CHCl_3_/MeOH/conc. NH_3_ 12:8:1); ^1^H NMR (500 MHz, CD_3_OD): *δ*=3.82 (dd, *J*=2.9, 11.1 Hz, 1H), 3.77 (ddd, *J*=4.7, 10.6, 11.0 Hz, 1H), 3.65 (dd, *J*=6.0, 11.1 Hz, 1H), 3.30–3.25 (m, 2H), 3.14 (dd, *J*=4.8, 12.4 Hz, 1H), 2.56–2.51 (m, 1H), 2.44 (dd, *J*=12.1, 12.1 Hz, 1H), 2.25–2.19 (m, 2H), 1.63–1.56 (m, 2H), 1.40–1.34 (m, 2H), 0.93 ppm (t, *J*=7.3 Hz, 3H); ^13^C NMR (125 MHz, CD_3_OD): *δ*=176.7, 77.3, 73.5, 62.7, 62.3, 53.2, 48.7, 36.9, 29.1, 23.3, 14.1 ppm; HRMS: *m*/*z* calcd: 247.1658 [*M*+H]^+^, found: 247.1651; elemental analysis calcd (%) for C_11_H_22_N_2_O_4_: C 53.64, H 9.00, N 11.37; found: C 53.57, H 9.03, N 11.29.

**2-Hexamido-1,5-imino-1,2,5-trideoxy-d-glucitol (23):** Yield: 48%; *R*_f_=0.55 (CHCl_3_/MeOH/conc. NH_3_ 12:8:1); ^1^H NMR (600 MHz, CD_3_OD): *δ*=3.81 (dd, *J*=3.1, 11.2 Hz, 1H), 3.74 (ddd, *J*=4.7, 11.0, 11.0 Hz, 1H), 3.64 (dd, *J*=4.1, 11.1 Hz, 1H), 3.28–3.23 (m, 2H), 3.11 (dd, *J*=4.7, 12.3 Hz, 1H), 2.50–2.47 (m, 1H), 2.40 (dd, *J*=11.9, 11.9 Hz, 1H), 2.25–2.19 (m, 2H), 1.64–1.59 (m, 2H), 1.37–1.29 (m, 4H), 0.90 ppm (t, *J*=6.8 Hz, 3H); ^13^C NMR (150 MHz, CD_3_OD): *δ*=176.7, 77.5, 73.8, 62.7, 62.6, 53.5, 48.9, 37.2, 32.5, 26.7, 23.4, 14.3 ppm; HRMS: *m*/*z* calcd: 261.1814 [*M*+H]^+^, found: 261.1807; elemental analysis calcd (%) for C_12_H_24_N_2_O_4_: C 55.36, H 9.29, N 10.76; found: C 55.31, H 9.25, N 10.68.

**1,5-Imino-2-isobutamido-1,2,5-trideoxy-d-glucitol (24):** Yield: 54%; *R*_f_=0.4 (CHCl_3_/MeOH/conc. NH_3_ 12:8:1); ^1^H NMR (500 MHz, CD_3_OD): *δ*=3.81 (dd, *J*=3.0, 11.2 Hz, 1H), 3.71 (ddd, *J*=4.8, 10.9, 10.9 Hz, 1H), 3.64 (dd, *J*=5.0, 11.2 Hz, 1H), 3.34–3.30 (m, 1H), 3.24 (dd, *J*=9.6, 9.6 Hz, 1H), 3.07 (dd, *J*=4.9, 12.4 Hz, 1H), 2.50–2.44 (m, 2H), 2.39 (dd, *J*=12.3, 12.3 Hz, 1H), 1.10 ppm (t, *J*=7.8 Hz, 3H); ^13^C NMR (125 MHz, CD_3_OD): *δ*=180.9, 77.5, 73.8, 62.8, 62.5, 53.6, 48.8, 36.4, 20.1, 19.8 ppm; HRMS: *m*/*z* calcd: 233.1501 [*M*+H]^+^, found: 233.1511; elemental analysis calcd (%) for C_10_H_20_N_2_O_4_: C 51.71, H 8.68, N 12.06; found: C 51.83, H 8.51, N 12.16.

**1,5-Imino-2-isovaleramido-1,2,5-trideoxy-d-glucitol (25):** Yield: 63%; *R*_f_=0.5 (CHCl_3_/MeOH/conc. NH_3_ 12:8:1); ^1^H NMR (600 MHz, CD_3_OD): *δ*=3.81–3.74 (m, 2H), 3.64 (dd, *J*=6.1, 10.5 Hz, 1H), 3.27–3.20 (m, 2H), 3.10 (dd, *J*=4.4, 12.3 Hz, 1H), 2.47–2.43 (m, 1H), 2.39 (dd, *J*=11.7, 11.7 Hz, 1H), 2.11–2.03 (m, 3H), 0.94 ppm (m, 6H); ^13^C NMR (150 MHz, CD_3_OD): *δ*=175.9, 77.7, 74.1, 62.9, 62.8, 53.8, 48.8, 46.5, 27.4, 22.8, 22.6 ppm; HRMS: *m*/*z* calcd: 247.1658 [*M*+H]^+^, found: 247.1661; elemental analysis calcd (%) for C_11_H_22_N_2_O_4_: C 53.64, H 9.00, N 11.37; found: C 53.59, H 8.98, N 11.31.

**Kinetic analysis of inhibitors:** Assays against O-GlcNAcase, *Vc*NagZ and *St*NagZ were performed in NaP_i_ buffer (50 mM, NaCl (100 mM), pH 6.5) and for β-hexosaminidase B citrate buffer (50 mM, NaCl (100 mM), pH 4.25) using 4-methylumbelliferyl *N*-acetyl-β-d-glucosaminide as substrate. For NAGLU, assays were performed in acetate buffer (100 mM, pH 4.3), containing bovine serum albumin (0.5 mgmL^−1^) using 4-methylumbelliferyl *N*-acetyl-α-d-glucosaminide as substrate. Release of 4-methylumbelliferone was monitored continuously using a fluorimeter plate reader for OGA, *Vc*NagZ and *St*NagZ, and a 30 min stopped-assay procedure was used for β-hexosaminidase B and NAGLU (quenched with fourfold excess of quenching buffer, glycine (200 mM), pH 10.75). For OGA, the inhibitors were preincubated with the enzyme for 10 min before the addition of the substrate. Readings were taken at excitation and emission wavelengths of 368 nm and 450 nm respectively. Assays contained substrate at the previously determined *K*_m_ value of the substrate for the enzyme, and the enzyme typically at a concentration of 100–200 nM. Inhibitors were added at a range of concentrations encompassing their *K*_i_ values. The rates at each inhibitor concentration were plotted and the best fit line through the points ascertained. The −1/*K*_i_ was taken as the point where the line of best fit intersected with 1/*V*_max_.

**NagZ crystallization, structure determination and refinement:**­ *St*NagZ plasmid construction, expression and purification have been previously described.^20^ *St*NagZ crystals were grown at RT using the hanging drop vapor-diffusion method by mixing equal volumes of reservoir buffer (25% PEG1000, MES (0.1M), pH 6.5) and protein solution (6 mgmL^−1^) in crystallization buffer (NaCl (150 mM), BisTris (20 mM), pH 6.5). A single *St*NagZ crystal was soaked overnight in a drop containing reservoir buffer and **21** at a concentration of 30 mM to obtain the protein–inhibitor complex. The 25% PEG1000 present in the buffer was sufficient for cryo-protection, and crystals were harvested by flash-cooling in liquid nitrogen. X-ray diffraction data were collected at beamline 08ID-1 at the Canadian Light Source (Saskatoon, Canada). Diffraction data were integrated using XDS^51^ and scaled and merged using SCALA^52^ (see Table S1). The structure was solved by molecular replacement using the program PHASER^53^ and the crystal structure of native unliganded *St*NagZ (PDB ID: 4GVG). Subsequent rounds of refinement were performed using phenix.refine and COOT.^54^,^55^ A ligand restraint file was generated for **21** using PHENIX eLBOW and the inhibitor was initially fit into electron density using PHENIX Ligandfit.^54^ Solvent molecules were added using phenix.refine and final refinement was performed using COOT and phenix.refine. Stereochemical quality of the final model was assessed by using MolProbity.^56^ The final refinement statistics are presented in Table S1.

**Determination of the Minimal Inhibitory Concentration of β-lactams:** Cultures were prepared by inoculating Mueller–Hinton broth (5 mL) with a small amount of a glycerol stock of *P. aeruginosa* PA01 and then were grown at 37°C to an OD_600_ value of ≈0.5. 96-well plates containing a range of concentrations of β-lactams varying by factors of 2 were prepared. Each well contained 80 μL of the antibiotic in Mueller–Hinton broth and the volume was made up to 100 μL by addition of either 20 μL of **21** (1 mM in H_2_O) or 20 μL H_2_O. These broths were inoculated with the culture (100 μL) and allowed to incubate at 37°C for 18 h. The optical density at 595 nm was measured for all cultures and the MIC determined from the concentration of antibiotic at which no growth was observed. All MIC determinations were performed in triplicate.

**Agar diffusion tests:** A culture of *P. aeruginosa* PA01 was prepared as described above. The cells were harvested by centrifugation (13000 rpm, 3 min). The cells were then resuspended in Mueller–Hinton broth (2 mL) and 500 μL of this suspension was used to inoculate the appropriate mixtures of inhibitor and Mueller–Hinton broth. Culture A contained Mueller–Hinton broth (500 μL) and **21** (500 μM in Mueller–Hinton broth, 500 μL). Culture B contained Mueller–Hinton broth (1000 μL). These mixtures were then cultured for 60 min at 37°C. Mueller–Hinton broth agar plates (1.5% agar) were streaked with the bacterial culture. Antibiotic discs (6 mm diameter) previously loaded with **21** (500 μM, 10 μL) or H_2_O alone, were placed on the agar plates. After incubation overnight at 37°C, the diameter of the inhibition zone was measured. All determinations were performed in triplicate.

## References

[1] Sanders CC (1987). Annu. Rev. Microbiol.

[2] Jacobs C, Huang LJ, Bartowsky E, Normark S, Park JT (1994). EMBO J.

[3] Jacoby G (2009). Clin. Microbiol. Rev.

[4] Jacoby GA, Munoz-Price LS (2005). N. Engl. J. Med.

[5] Reisbig MD, Hanson ND (2002). J. Antimicrob. Chemother.

[6] Bradford PA, Urban C, Mariano N, Projan SJ, Rahal JJ, Bush K (1997). Antimicrob. Agents Chemother.

[7] Tondi D, Morandi F, Bonnet R, Costi MP, Shoichet BK (2005). J. Am. Chem. Soc.

[8] Eidam O, Romagnoli C, Dalmasso G, Barelier S, Caselli E, Bonnet R, Shoichet BK, Prati F (2012). Proc. Natl. Acad. Sci. USA.

[9] Fisher JF, Meroueh SO, Mobashery S (2005). Chem. Rev.

[10] Vollmer W, Holtje JV (2001). Curr. Opin. Microbiol.

[11] Park JT (1993). J. Bacteriol.

[12] Cheng Q, Li H, Merdek K, Park JT (2000). J. Bacteriol.

[13] Votsch W, Templin MF (2000). J. Biol. Chem.

[14] Jacobs C, Frere J-M, Normark S (1997). Cell.

[15] Uehara T, Park JT (2002). J. Bacteriol.

[16] Henrissat B, Davies G (1997). Curr. Opin. Struct. Biol.

[17] Vocadlo DJ, Mayer C, He S, Withers SG (2000). Biochemistry.

[18] Vocadlo DJ, Withers SG (2005). Biochemistry.

[19] Stubbs KA, Scaffidi A, Debowski AW, Mark BL, Stick RV, Vocadlo DJ (2008). J. Am. Chem. Soc.

[20] Bacik J-P, Whitworth GE, Stubbs KA, Vocadlo DJ, Mark BL (2012). Chem. Biol.

[21] Stubbs KA, Balcewich M, Mark BL, Vocadlo DJ (2007). J. Biol. Chem.

[22] Yamaguchi T, Blázquez B, Hesek D, Lee M, Llarrull LI, Boggess B, Oliver AG, Fisher JF, Mobashery S (2012). ACS Med. Chem. Lett.

[23] Asgarali A, Stubbs KA, Oliver A, Vocadlo DJ, Mark BL (2009). Antimicrob. Agents Chemother.

[24] Zamorano L, Reeve TM, Deng L, Juan C, Moya B, Cabot G, Vocadlo DJ, Mark BL, Oliver A (2010). Antimicrob. Agents Chemother.

[25] Huang Y-W, Hu R-M, Lin C-W, Chung T-C, Yang T-C (2012). Antimicrob. Agents Chemother.

[26] Balcewich M, Stubbs KA, He Y, James T, Davies GJ, Vocadlo DJ, Mark BL (2009). Protein Sci.

[27] Beer D, Maloisel JL, Rast DM, Vasella A (1990). Helv. Chim. Acta.

[28] Dong DL, Hart GW (1994). J. Biol. Chem.

[29] Haltiwanger RS, Grove K, Philipsberg GA (1998). J. Biol. Chem.

[30] Miller DJ, Gong X, Shur BD (1993). Development.

[31] Ficko-Blean E, Stubbs KA, Nemirovsky O, Vocadlo DJ, Boraston AB (2008). Proc. Natl. Acad. Sci. USA.

[32] Gloster TM, Vocadlo DJ (2012). Nat. Chem. Biol.

[33] Stubbs KA, Zhang N, Vocadlo DJ (2006). Org. Biomol. Chem.

[34] Tropak MB, Reid SP, Guiral M, Withers SG, Mahuran D (2004). J. Biol. Chem.

[35] Ho CW, Popat SD, Liu TW, Tsai KC, Ho MJ, Chen WH, Yang AS, Lin CH (2010). ACS Chem. Biol.

[36] Fleet GWJ, Smith PW, Nash RJ, Fellows LE, Parekh RB, Rademacher TW (1986). Chem. Lett.

[37] Fleet GWJ, Fellows LE, Smith PW (1987). Tetrahedron.

[38] Kappes E, Legler G (1989). J. Carbohydr. Chem.

[39] Furneaux RH, Gainsford GJ, Lynch GP, Yorke SC (1993). Tetrahedron.

[40] Granier T, Vasella A (1998). Helv. Chim. Acta.

[41] Steiner AJ, Schitter G, Stutz AE, Wrondigg TM, Tarling CA, Withers SG, Mahuran DJ, Tropak MJ (2009). Tetrahedron: Asymmetry.

[42] Gandy MN, Piggott MJ, Stubbs KA (2010). Aust. J. Chem.

[43] Billing JF, Nilsson UJ (2005). Tetrahedron.

[44] Goddard-Borger ED, Stick RV (2007). Org. Lett.

[45] Nilsson KGI, Pan H, Larsson-Lorek U (1997). J. Carbohydr. Chem.

[46] Zhao K-W, Neufeld EF (2000). Protein Expression Purif.

[47] Sanders CC, Sanders WE (1992). Clin. Infect. Dis.

[48] Govan JR, Deretic V (1996). Microbiol. Rev.

[49] Lyczak JB, Cannon CL, Pier GB (2002). Clin. Microbiol. Rev.

[50] Nagaki M, Shimura S, Tanno Y, Ishibashi T, Sasaki H, Takishima T (1992). Chest.

[51] Kabsch W (2010). Acta Crystallogr. D Biol. Crystallogr.

[52] Evans P (2006). Acta Crystallogr. D Biol. Crystallogr.

[53] McCoy AJ, Grosse-Kunstleve RW, Storoni LC, Read RJ (2005). Acta Crystallogr. D Biol. Crystallogr.

[54] Adams PD, Afonine PV, Bunkoczi G, Chen VB, Davis IW, Echols N, Headd JJ, Hung LW, Kapral GJ, Grosse-Kunstleve RW, McCoy AJ, Moriarty NW, Oeffner R, Read RJ, Richardson DC, Richardson JS, Terwilliger TC, Zwart PH (2010). Acta Crystallogr. D Biol. Crystallogr.

[55] Emsley P, Cowtan K (2004). Acta Crystallogr. D Biol. Crystallogr.

[56] Chen VB, Arendall WB, Headd JJ, Keedy DA, Immormino RM, Kapral GJ, Murray LW, Richardson JS, Richardson DC (2010). Acta Crystallogr. D Biol. Crystallogr.

