# Supplementary material for: The Development of Selective Inhibitors of NagZ: Increased Susceptibility of Gram-Negative Bacteria to β-Lactams
Source: Chembiochem. 2013 Sep 5;14(15):1973–81. doi: 10.1002/cbic.201300395 (PMC3920638; doi:10.1002/cbic.201300395)
Supplement: Supplementary file 1 [file cbic0014-1973-sd1.pdf]

CHEMBIOCHEM

# Supporting Information

© Copyright Wiley-VCH Verlag GmbH & Co. KGaA, 69451 Weinheim, 2013

## The Development of Selective Inhibitors of NagZ: Increased Susceptibility of Gram-Negative Bacteria to β-Lactams

Keith A. Stubbs,*[a] John-Paul Bacik,[b, d] G. Evan Perley-Robertson,[c] Garrett E. Whitworth,[c, e] Tracey M. Gloster,[c, f] David J. Vocadlo,[c] and Brian L. Mark*[b]

cbic_201300395_sm_miscellaneous_information.pdf

## Table S1: Crystallographic data and model refinement

| | ***St*NagZ-21 (PDB:4HZM)** |
|---|---|
| **Data Collection**[a] | |
| Space group | P1 $2_1$ 1 |
| Unit cell (Å) | a=49.77, b=66.19, c=95.07 |
| | $\alpha$ = 90, $\beta$ = 99.21, $\gamma$ = 90º |
| Wavelength (Å) | 0.9795 |
| Resolution range (Å) | 49.12 – 1.45 (1.53 – 1.45) |
| Total observations | 378412 |
| Total unique observations | 105765 |
| $I/\sigma_I$ | 13.0 (3.1) |
| Completeness (%) | 98.0 (87.5) |
| $R_{merge}$ | 0.065 (0.365) |
| Redundancy | 3.6 (2.5) |
| **Refinement Statistics** | |
| Resolution (Å) | 49.12 – 1.45 |
| Reflections (total) | 105697 |
| Reflections (test) | 3160 |
| Total atoms refined | 6089 |
| Solvent | 920 |
| $R_{work}$ ($R_{free}$) | 0.169 (0.193) |
| Mean B values (Å$^2$) | 14.5 |
| Overall | |
| Bound ligand | 7.9/7.9 |
| (A/B subunit) | |
| RMSDs | 0.008/1.172 |
| Bond lengths (Å) / angles (º) | |
| Ramachandran plot | 97.1/2.9 |
| Favored/allowed (%) | |

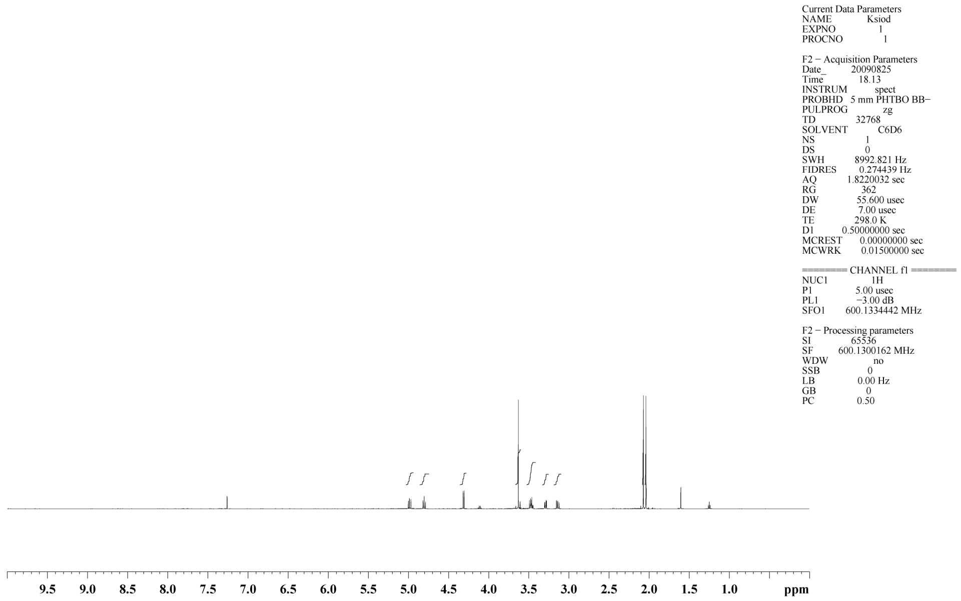


$^{1}$H NMR of **5**.

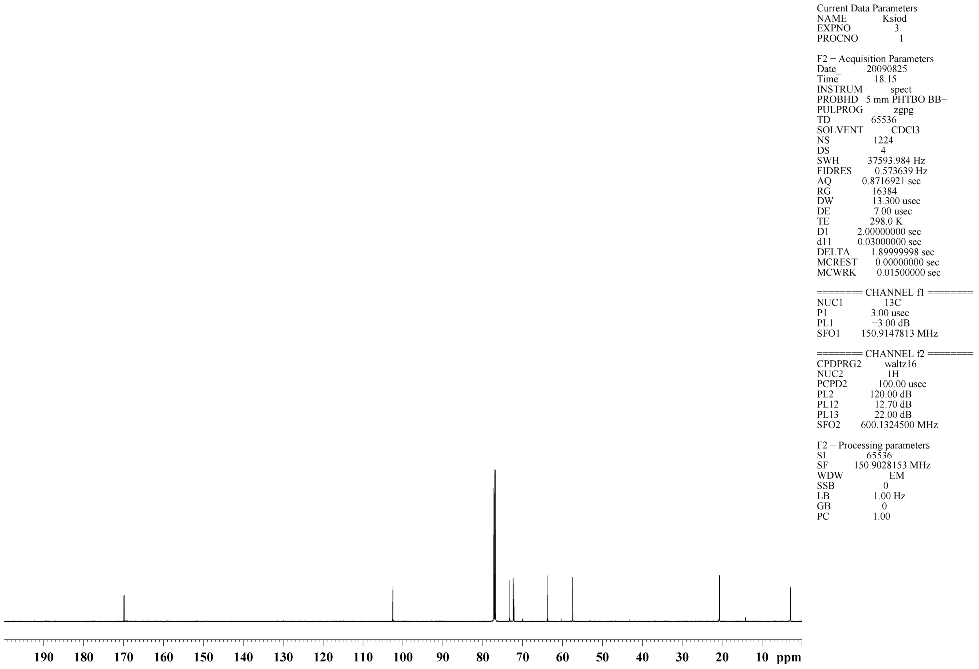


$^{13}$C NMR of **5**.

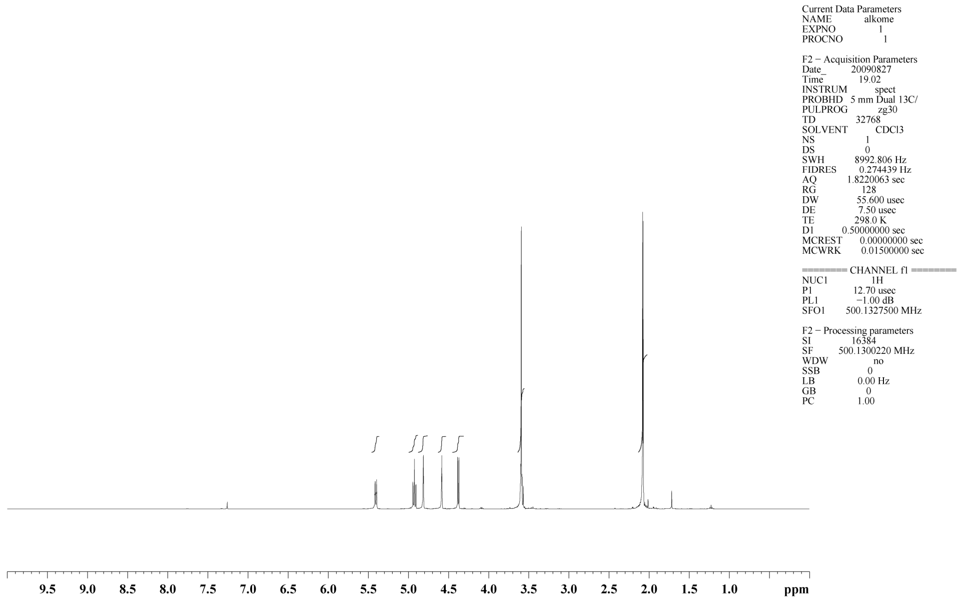


$^{1}$H NMR of **2**.

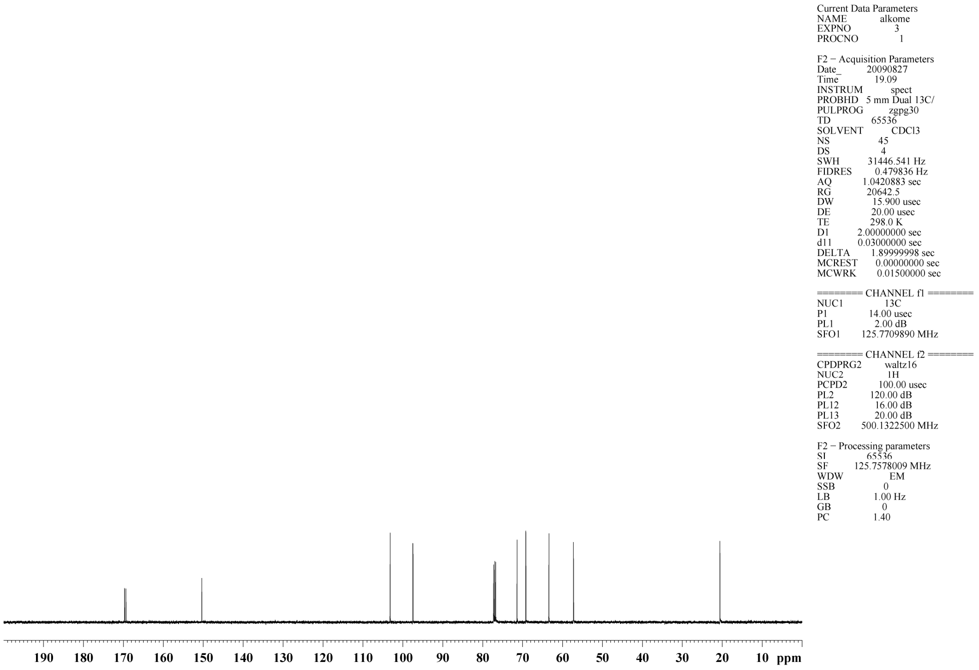


$^{13}$C NMR of **2**.

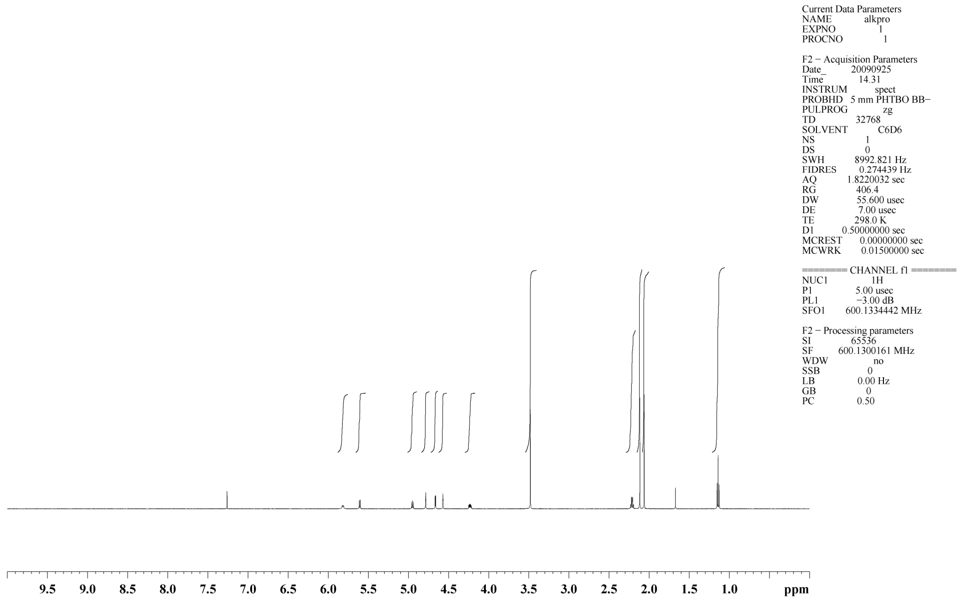


$^{1}$H NMR of **7**.

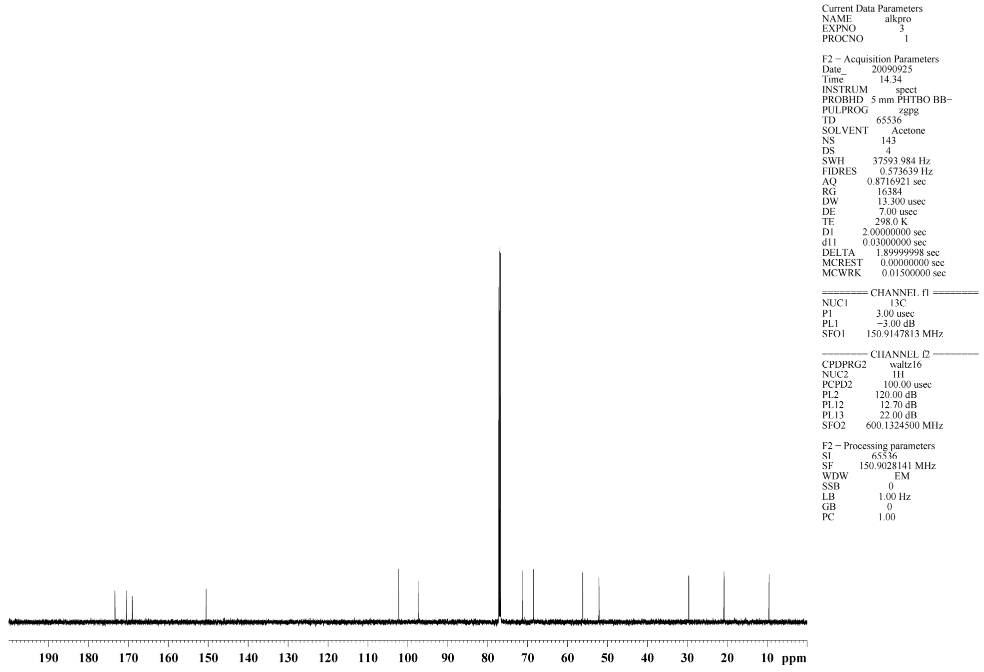


$^{13}$C NMR of **7**.

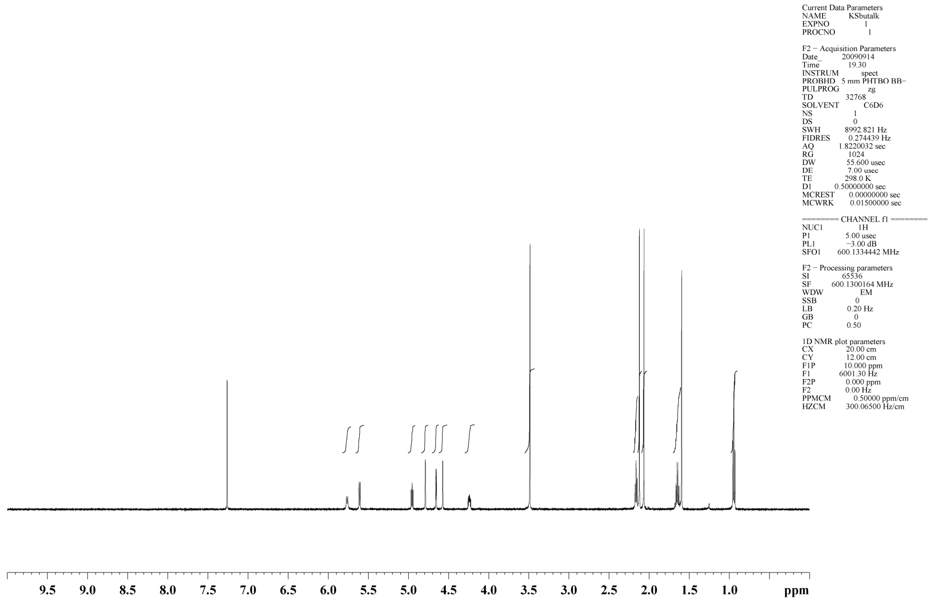


$^{1}$H NMR of **8**.

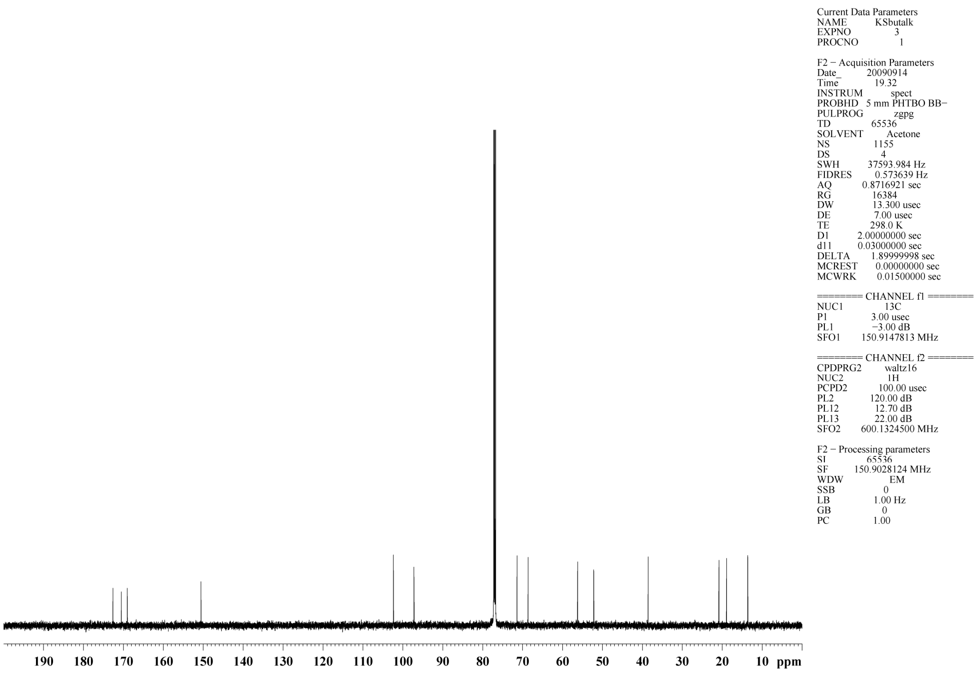


$^{13}$C NMR of **8**.

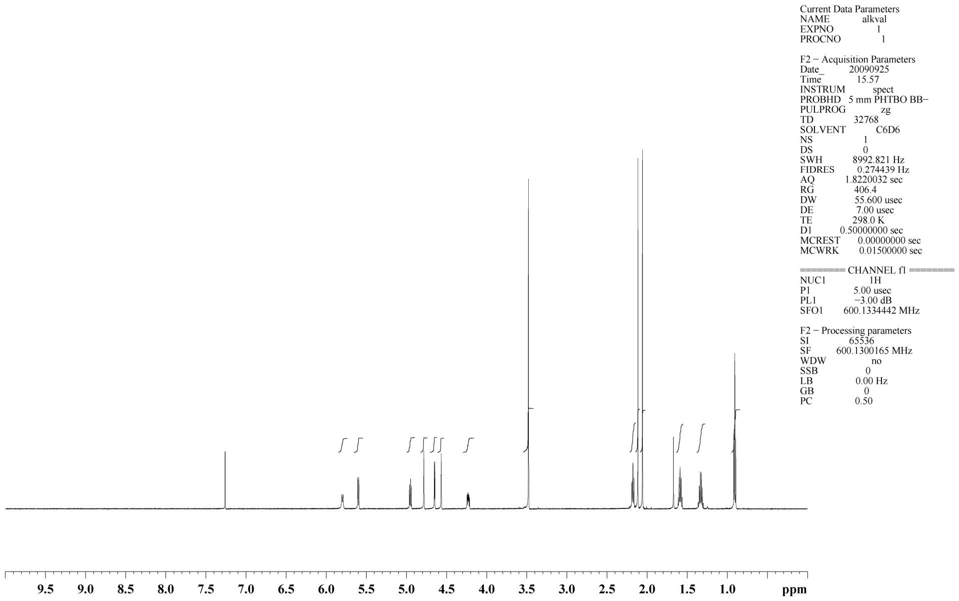


$^{1}$H NMR of **9**.

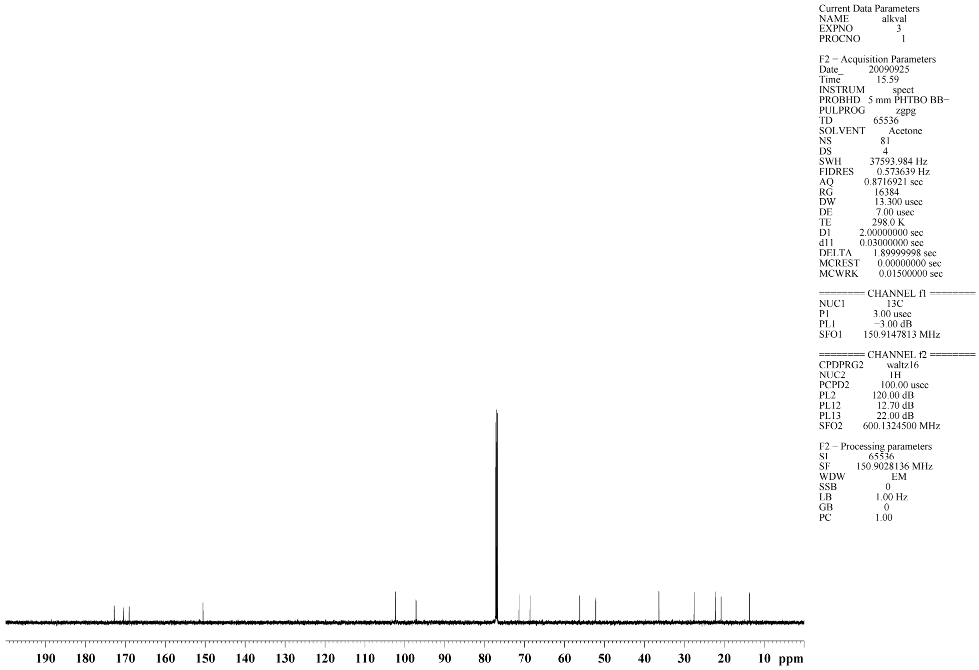


$^{13}$C NMR of **9**.

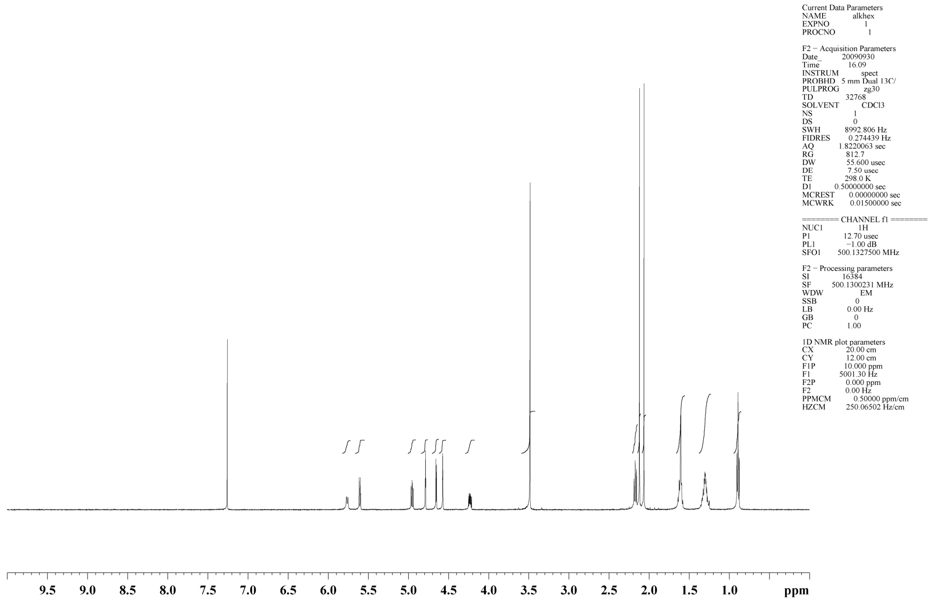


$^1$H NMR of **10**.

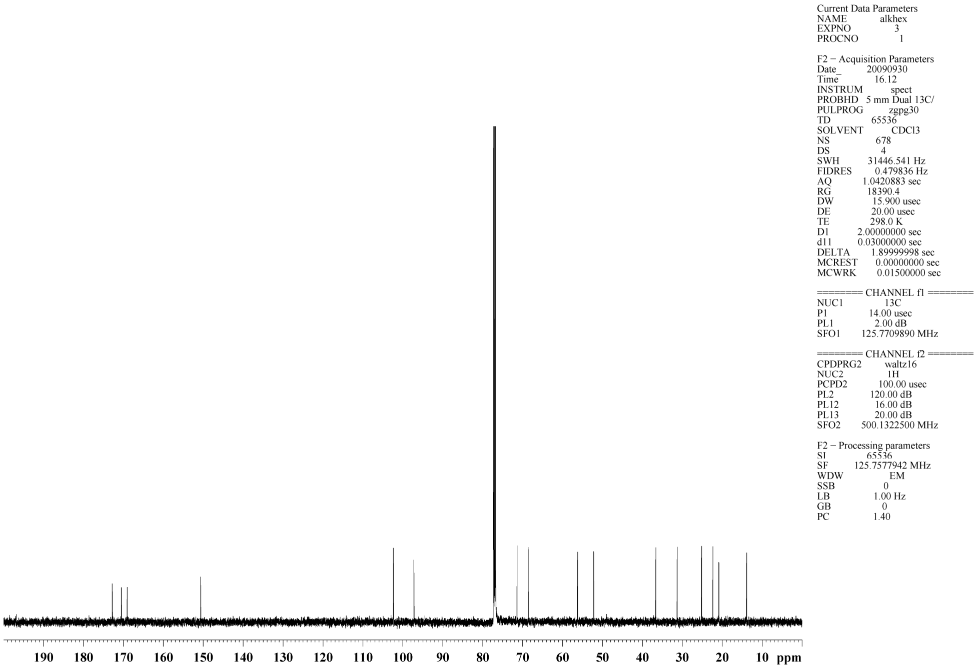


$^{13}$C NMR of **10**.

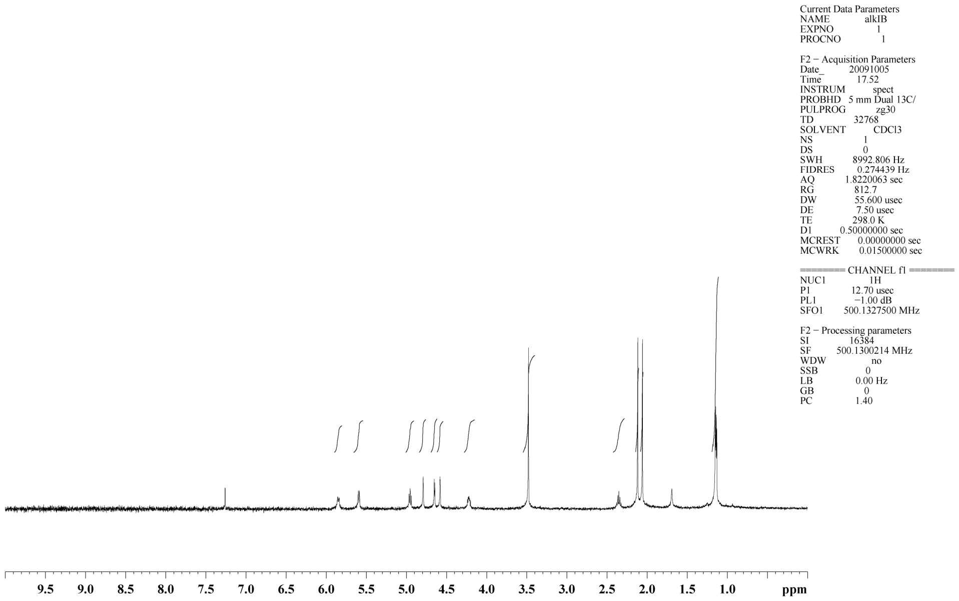


$^{1}$H NMR of **11**.

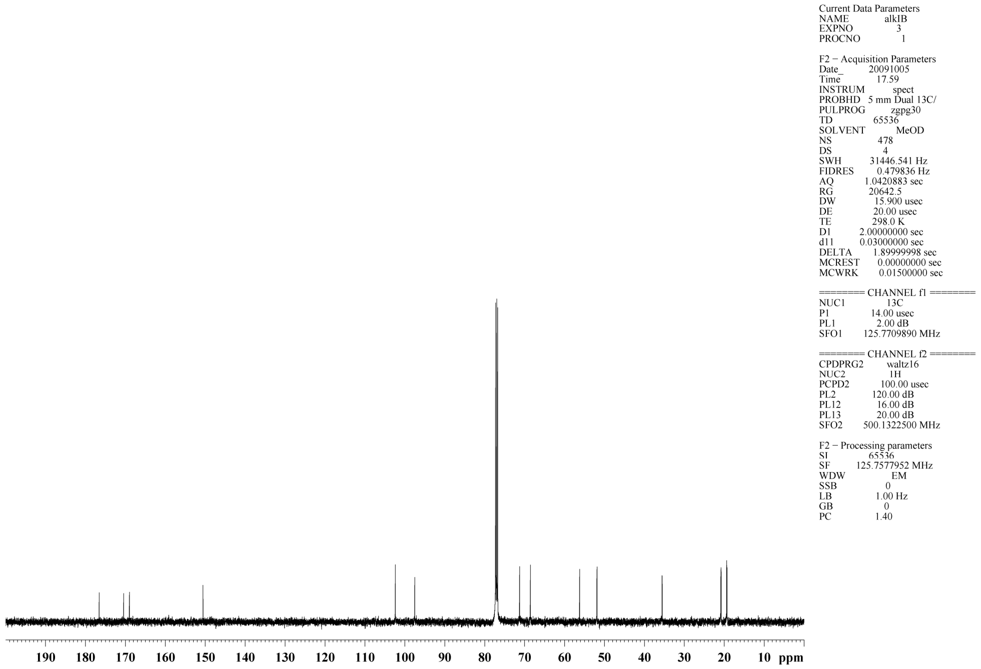


$^{13}$C NMR of **11**.

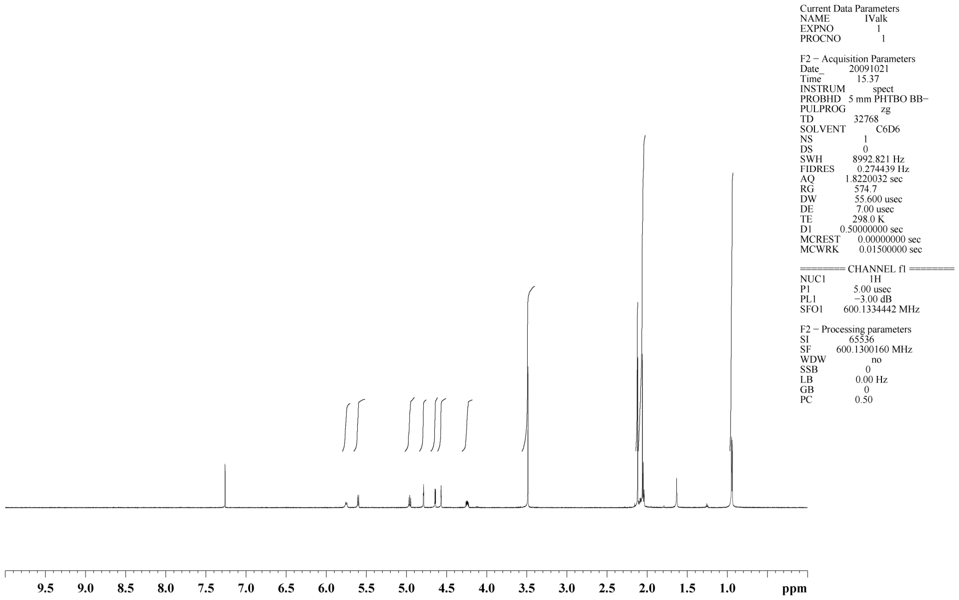


$^{1}$H NMR of **12**.

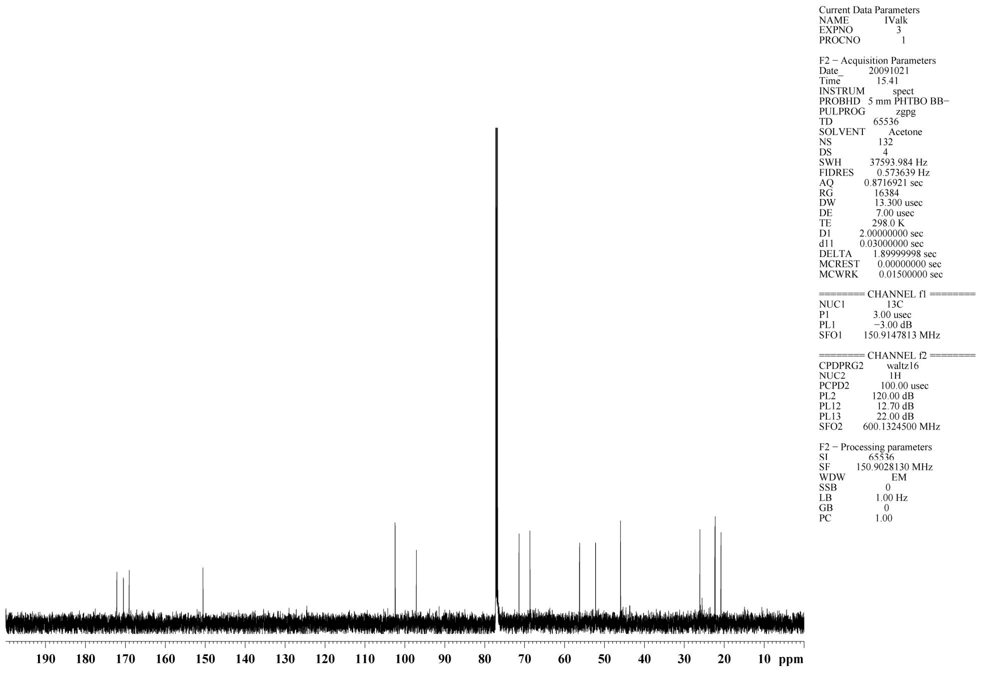


$^{13}$C NMR of **12**.

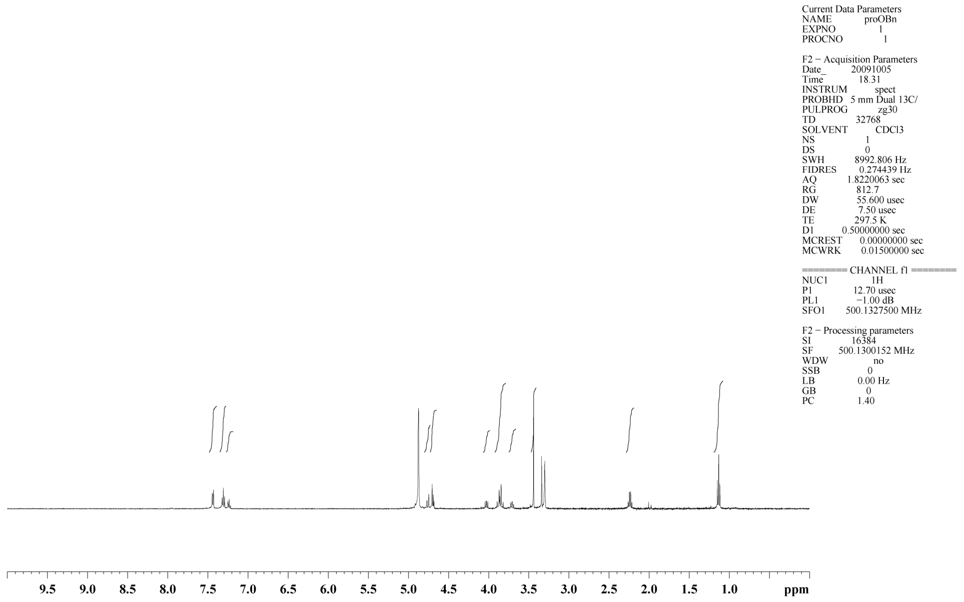


$^{1}$H NMR of **14**.

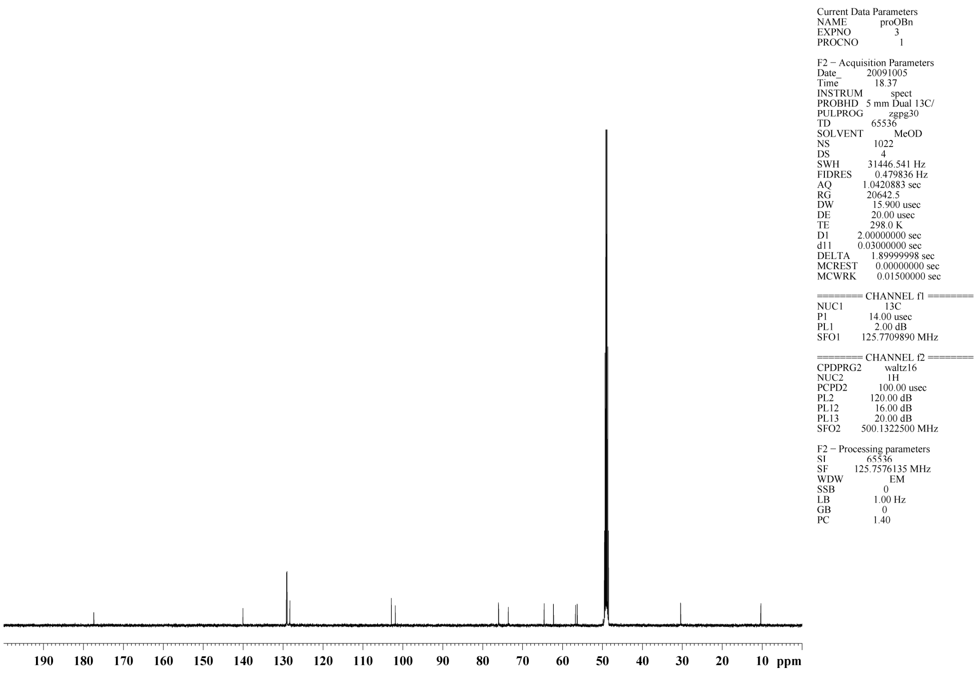


$^{13}$C NMR of **14**.

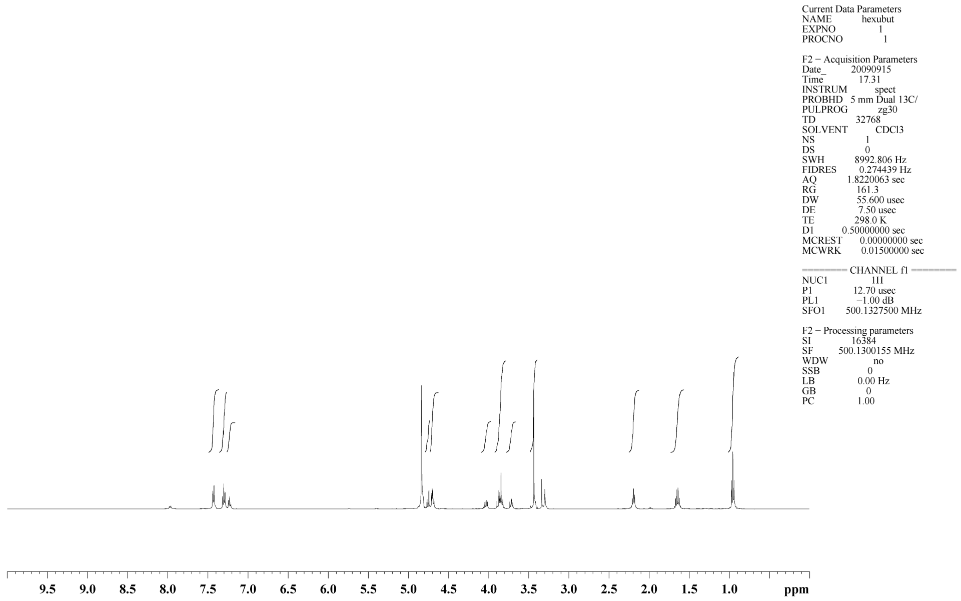


$^{1}$H NMR of **15**.

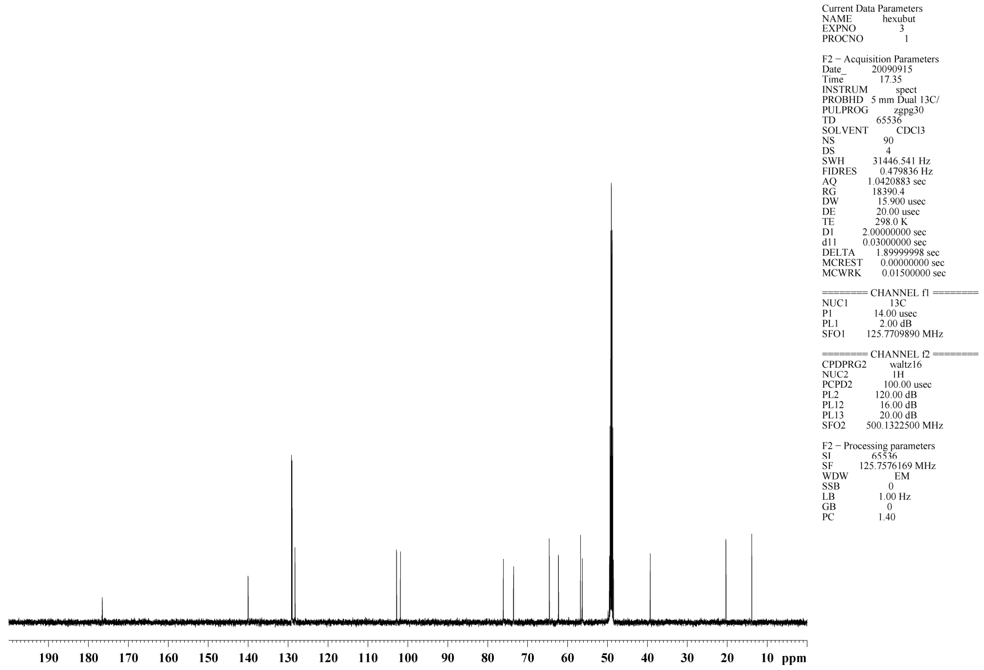


$^{13}$C NMR of **15**.

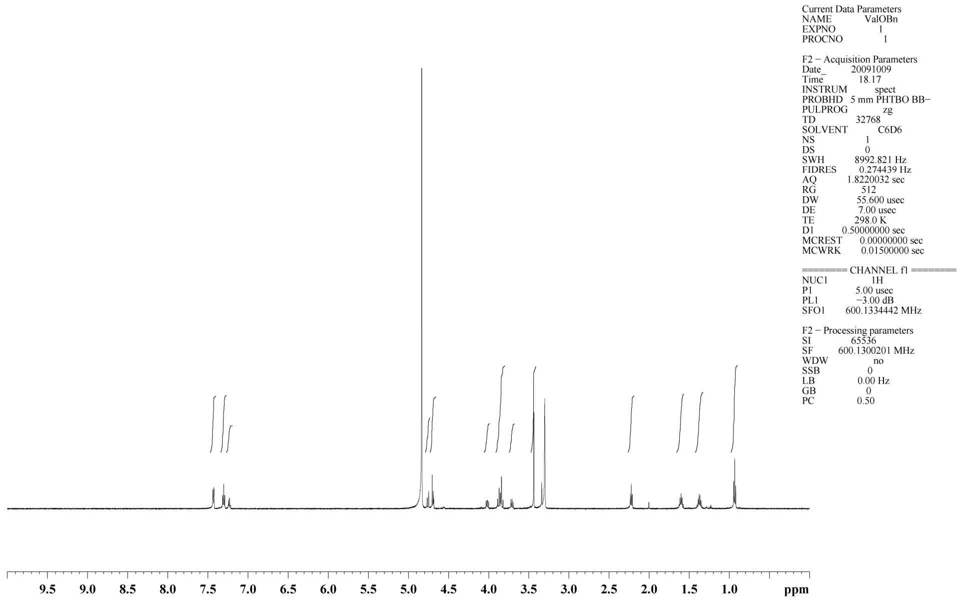


$^{1}$H NMR of **16**.

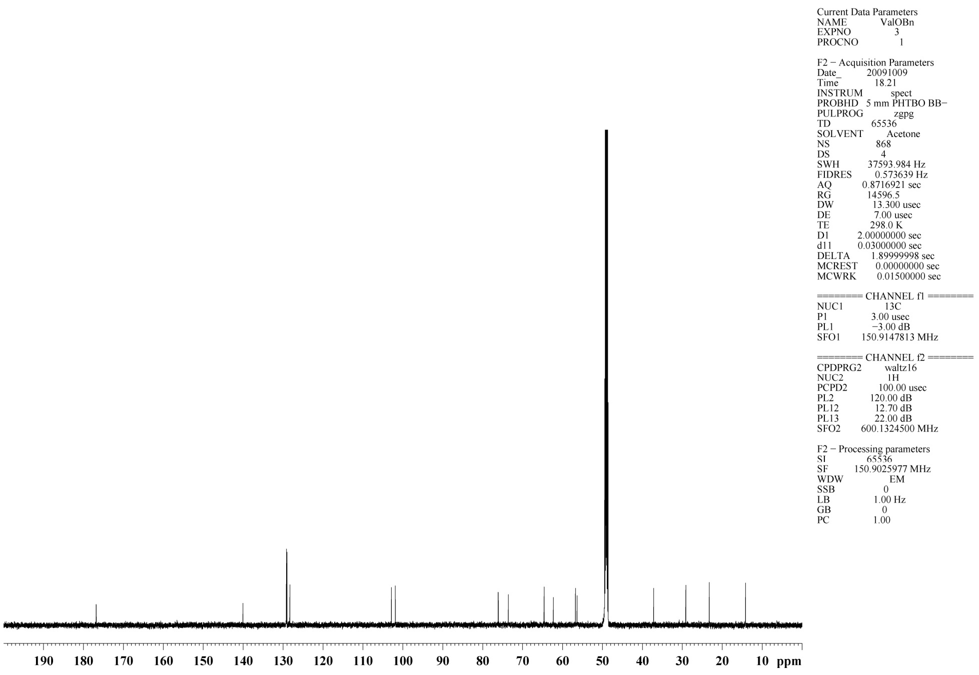


$^{13}$C NMR of **16**.

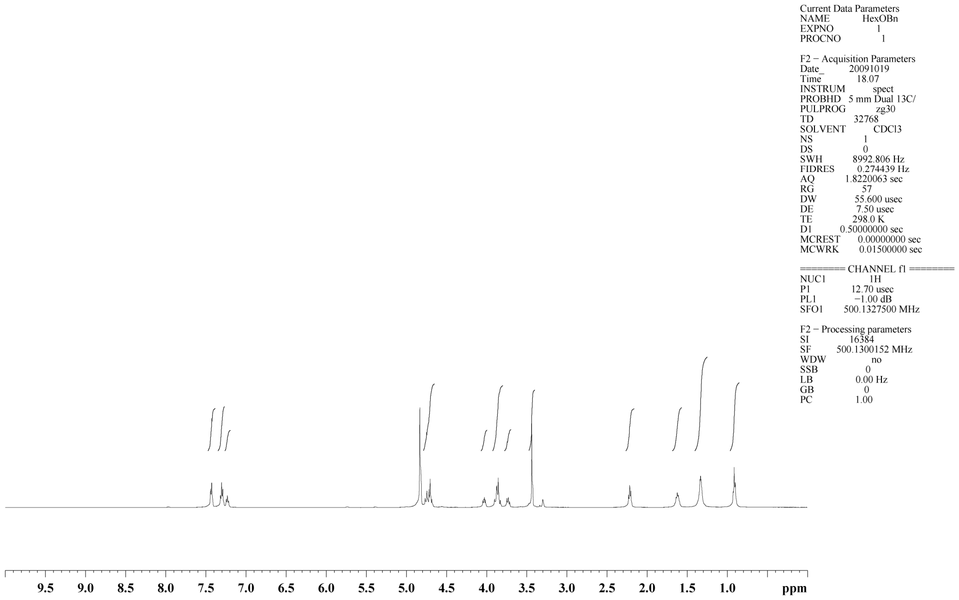


$^1$H NMR of **17**.

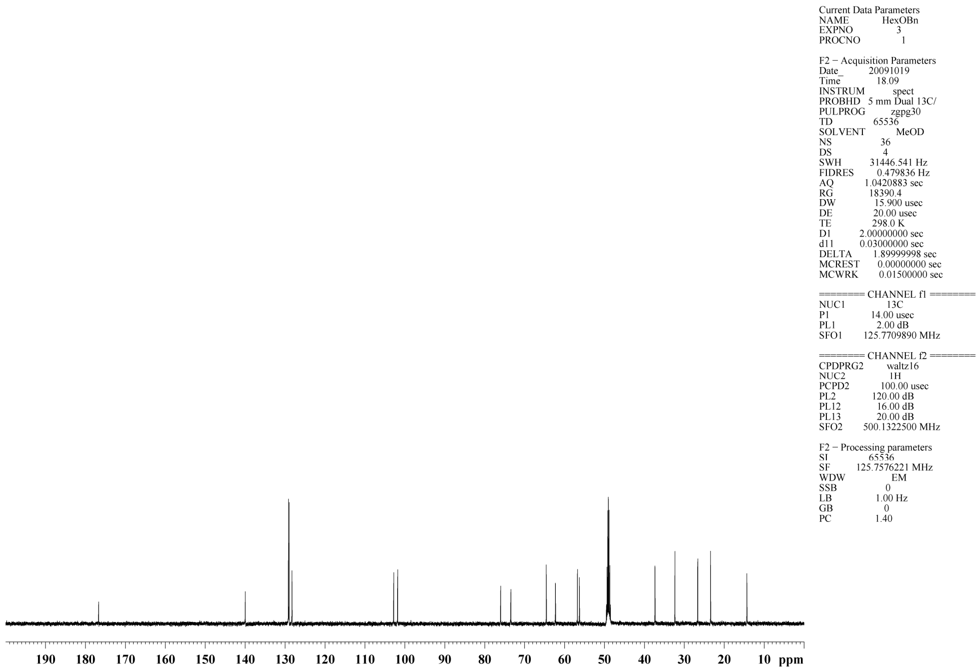


$^{13}$C NMR of **17**.

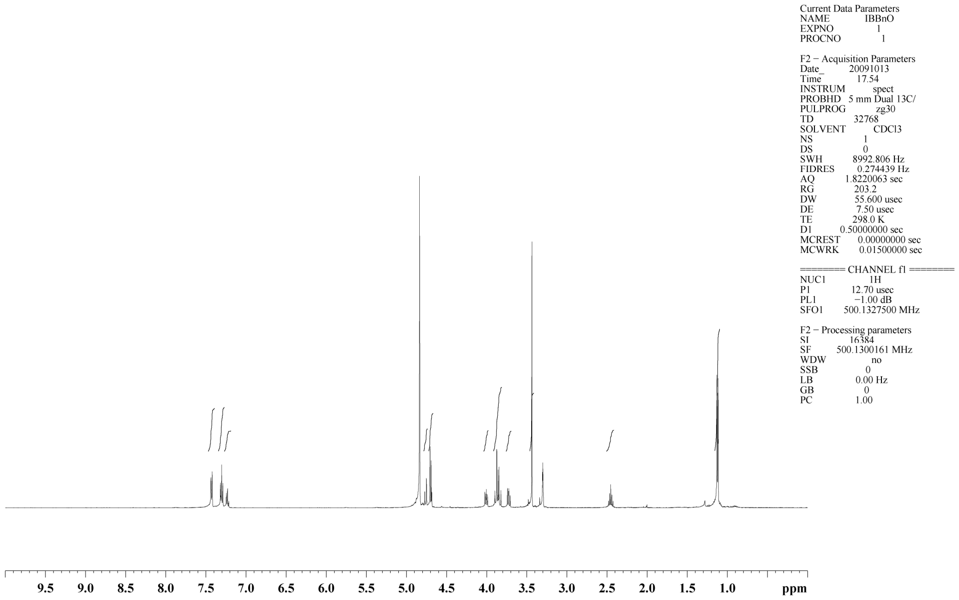


$^1$H NMR of **18**.

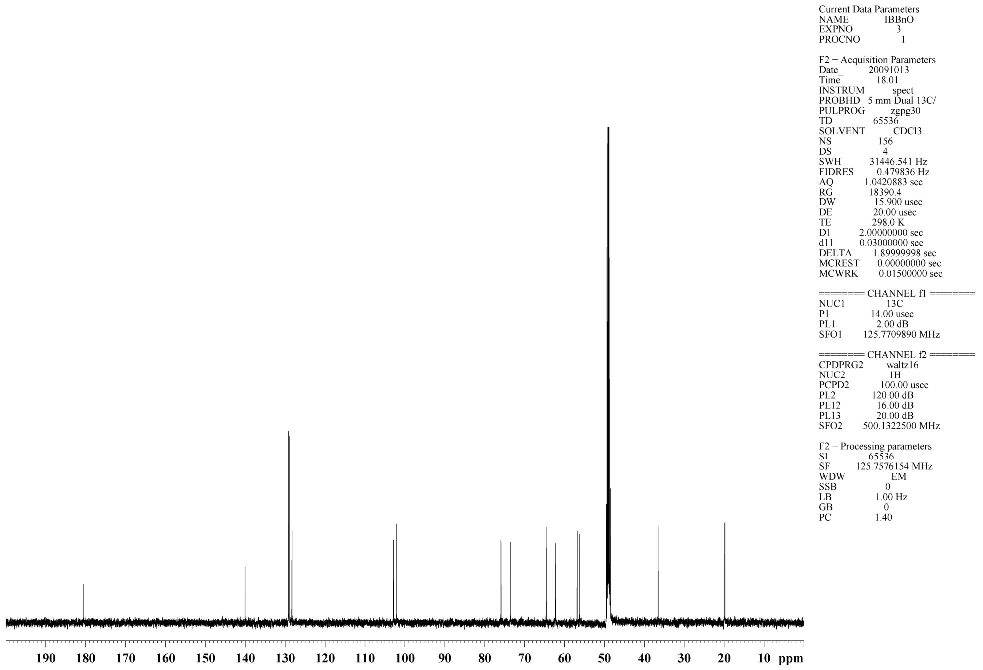


$^{13}$C NMR of **18**.

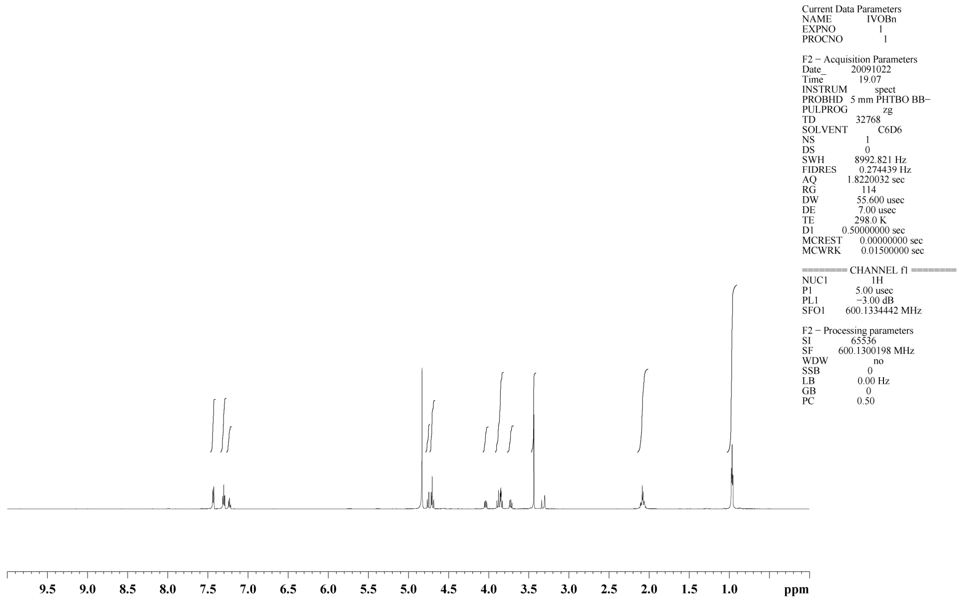


$^{1}$H NMR of **19**.

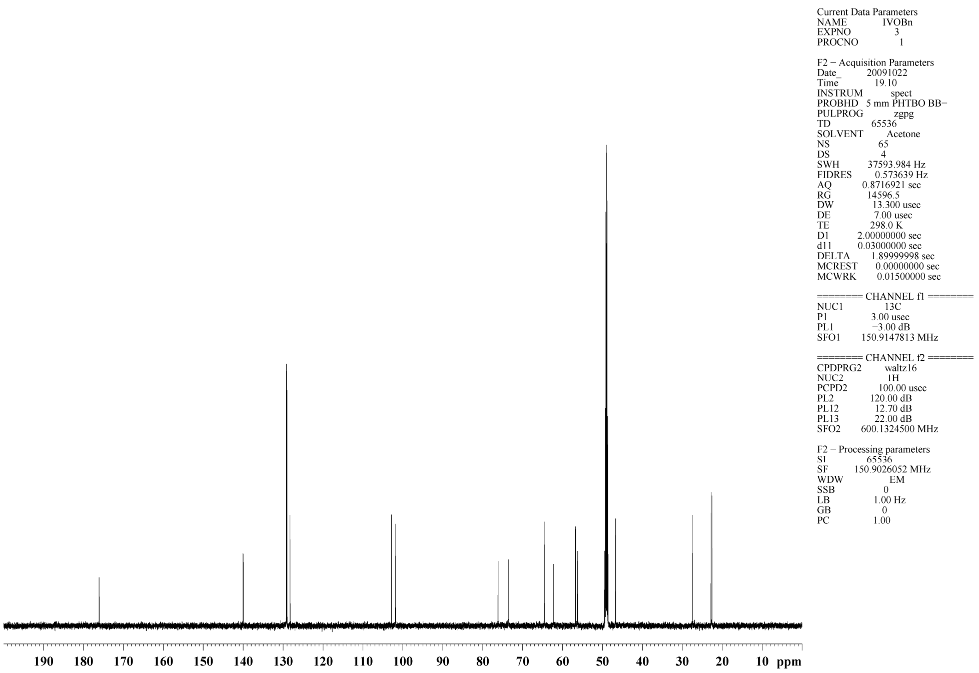


$^{13}$C NMR of **19**.

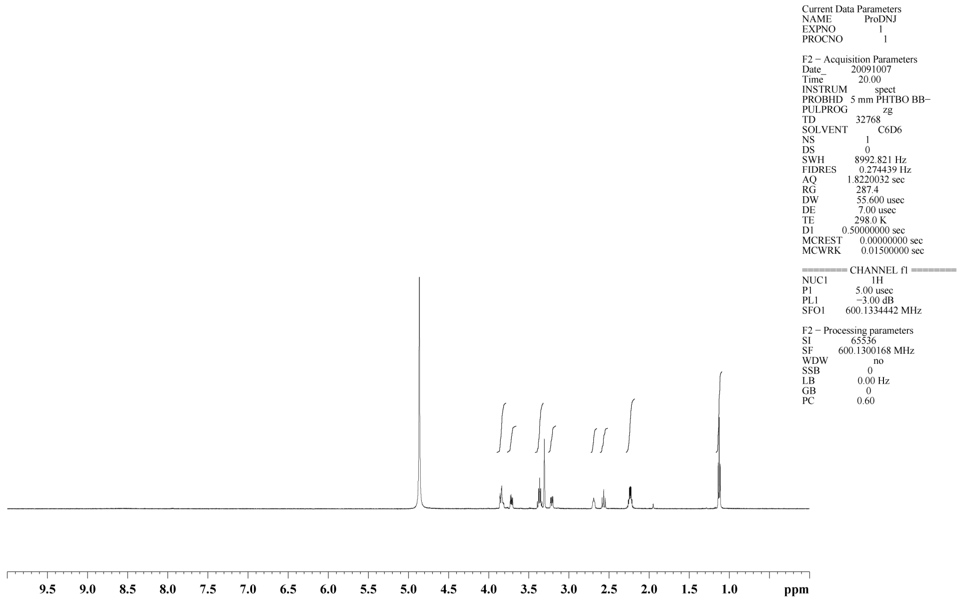


$^{1}$H NMR of **20**.

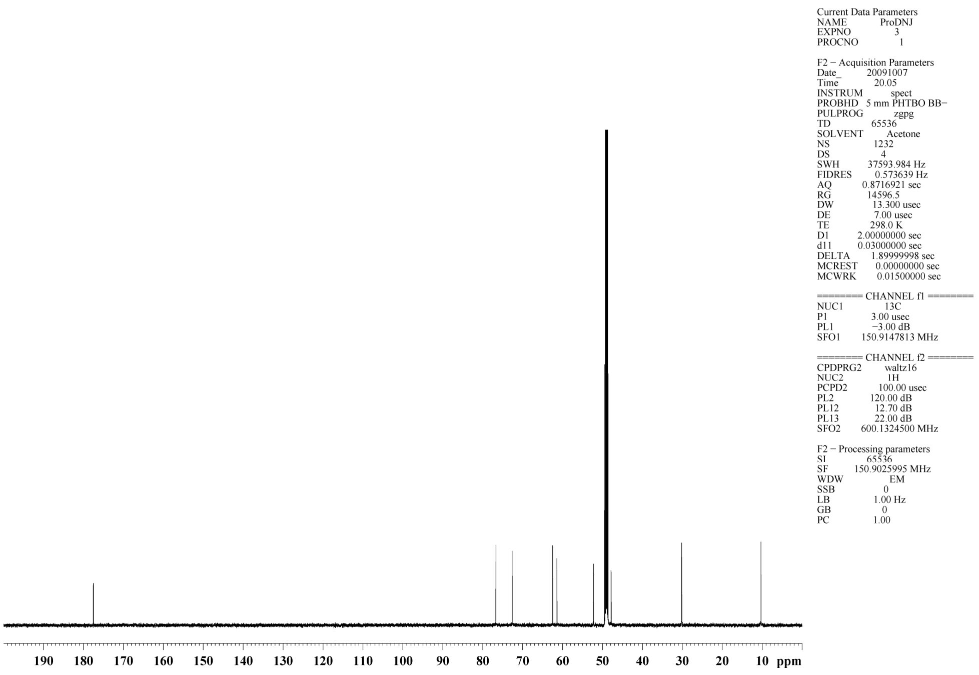


$^{13}$C NMR of **20**.

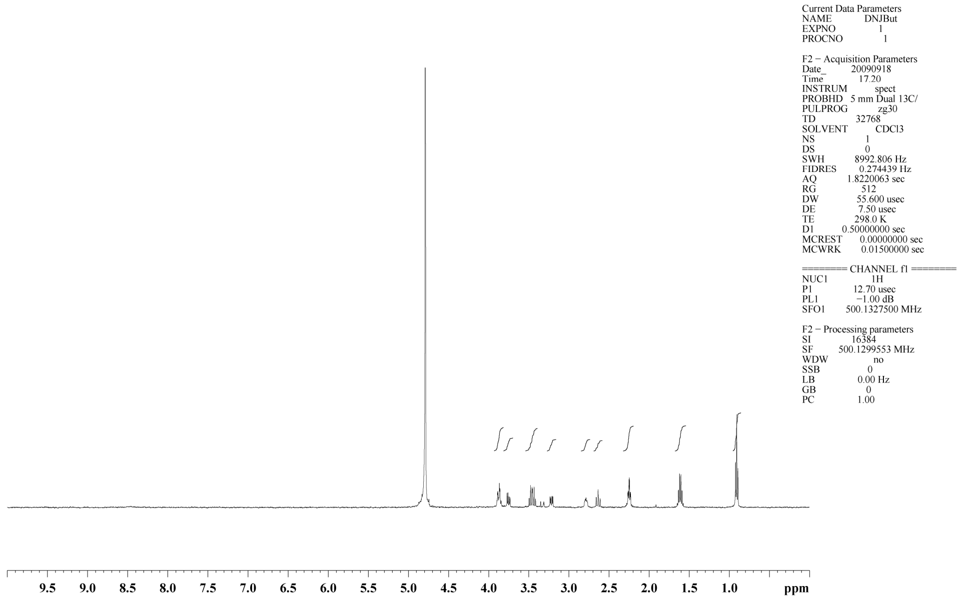


$^1$H NMR of **21**.

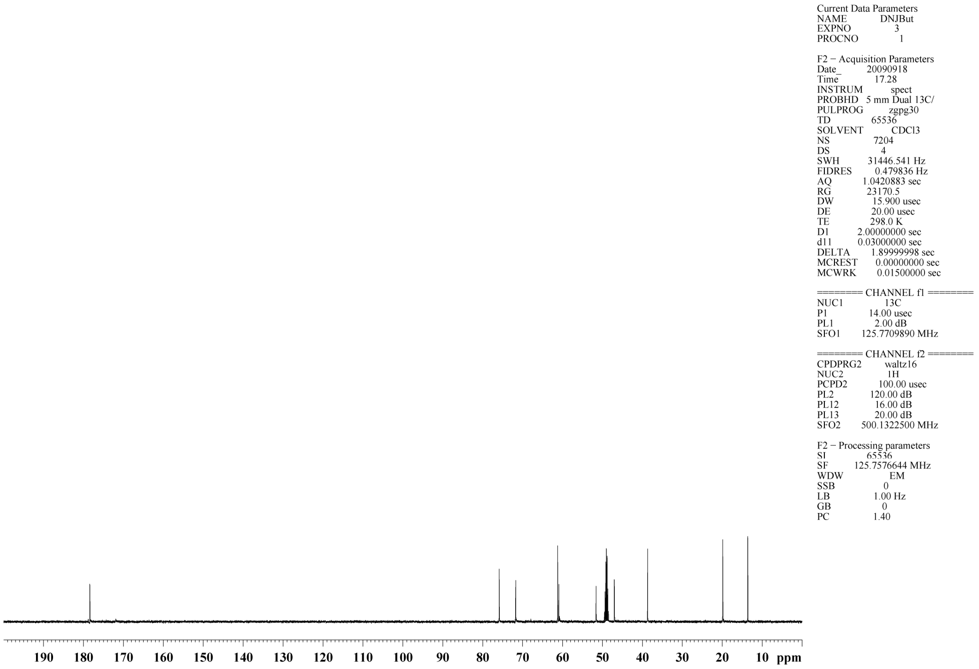


$^{13}$C NMR of **21**.

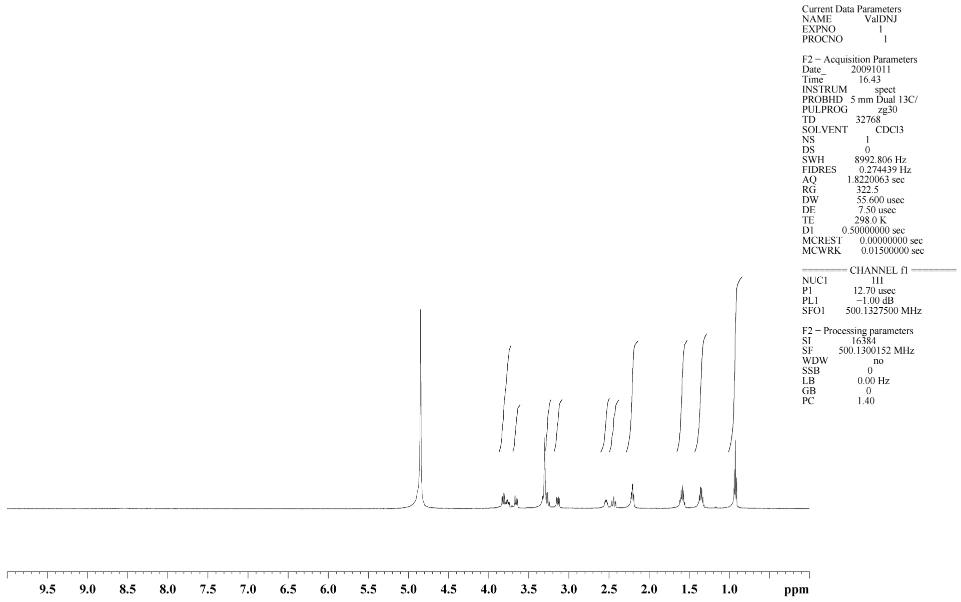


$^{1}$H NMR of **22**.

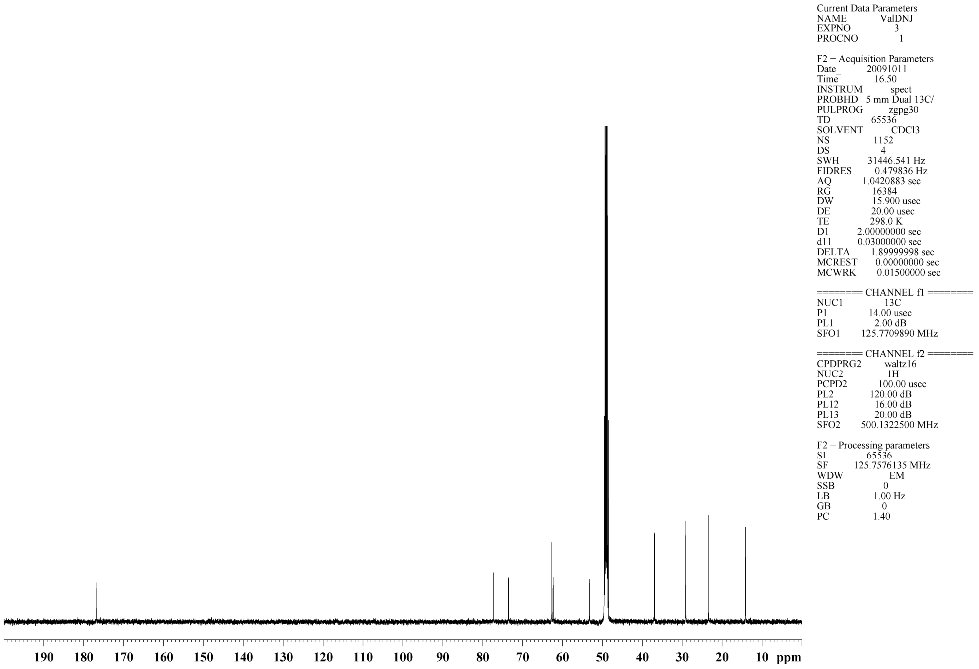


$^{13}$C NMR of **22**.

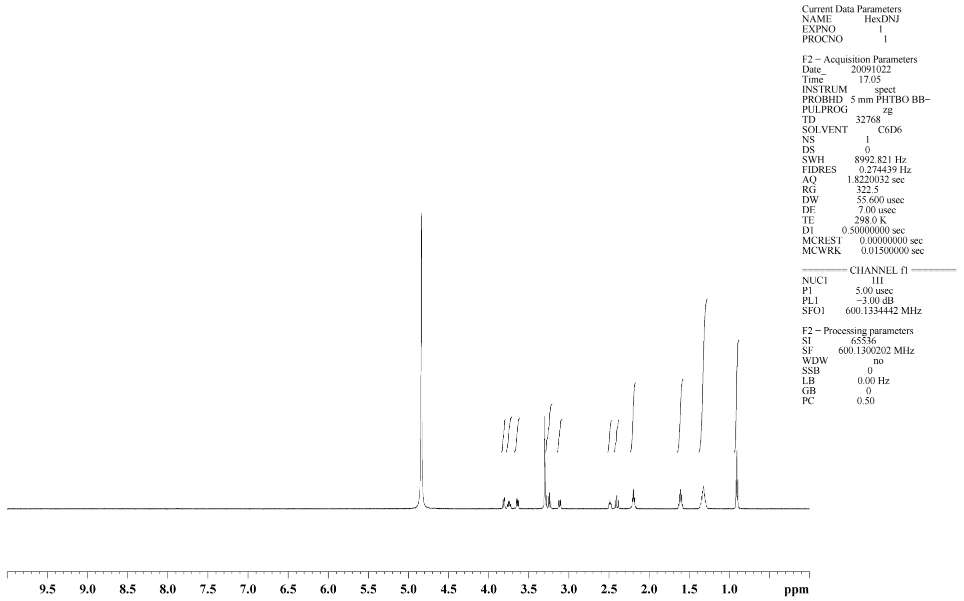


$^{1}$H NMR of **23**.

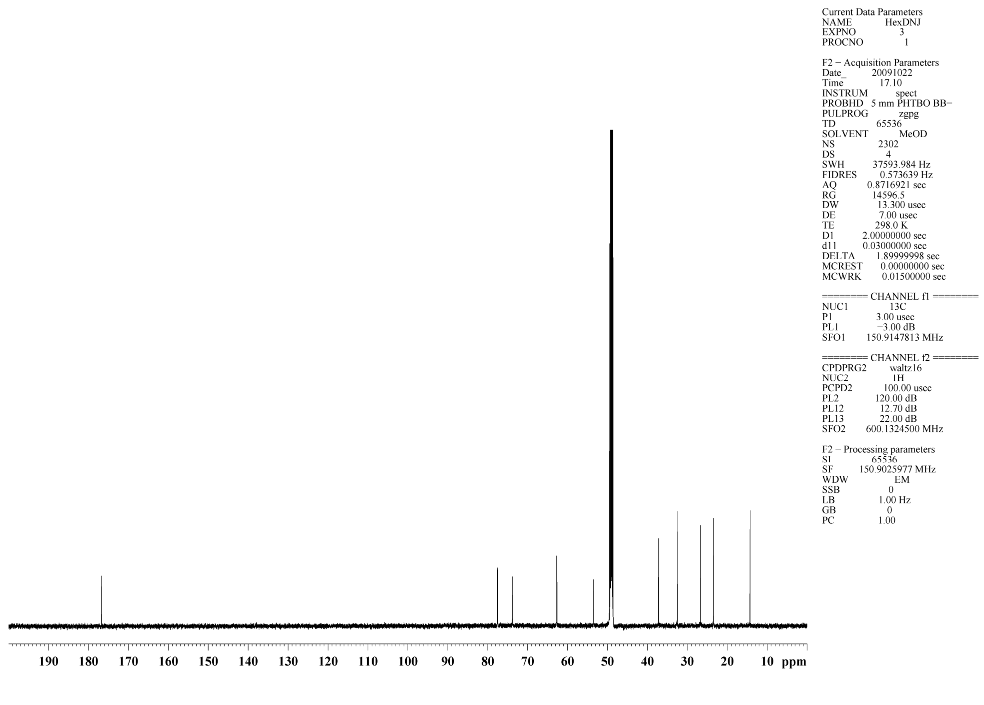


$^{13}$C NMR of **23**.

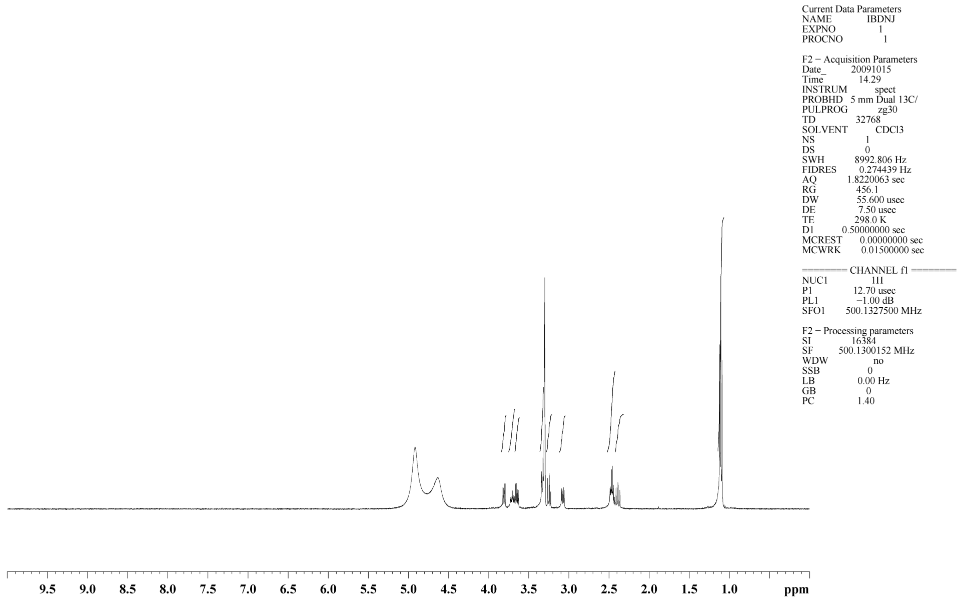


$^{1}$H NMR of **24**.

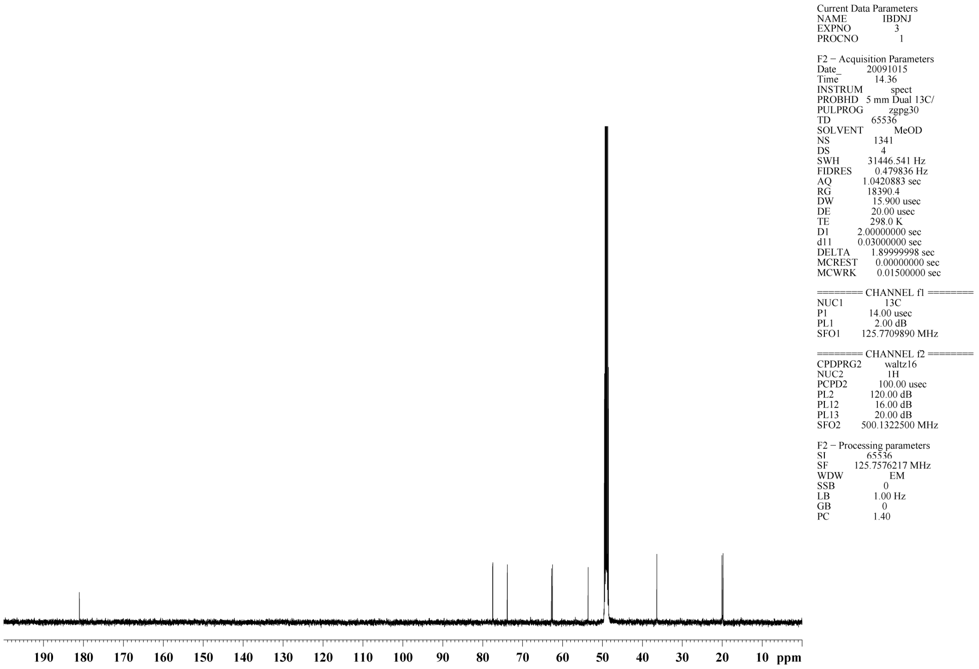


$^{13}$C NMR of **24**.

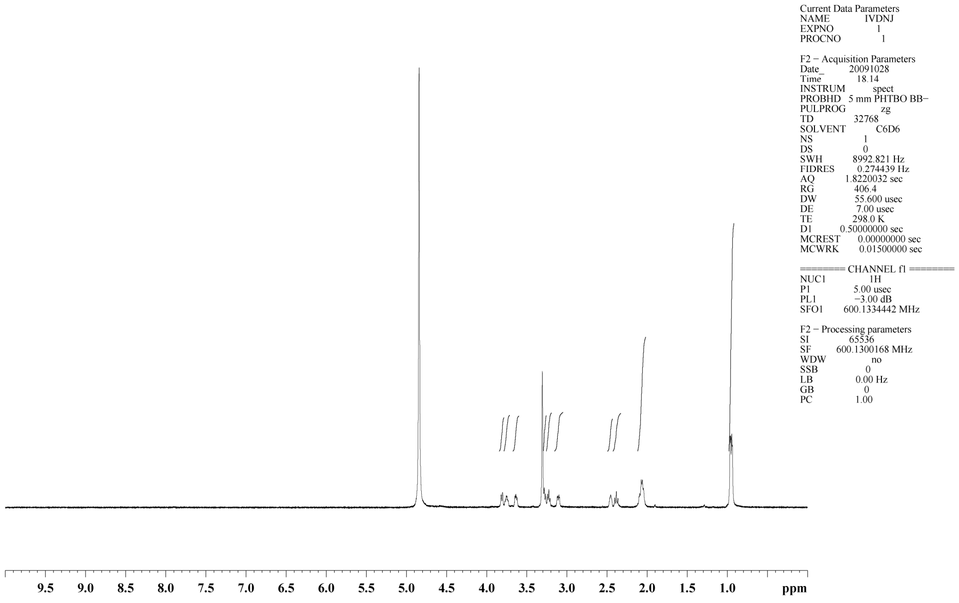


$^{1}$H NMR of **25**.

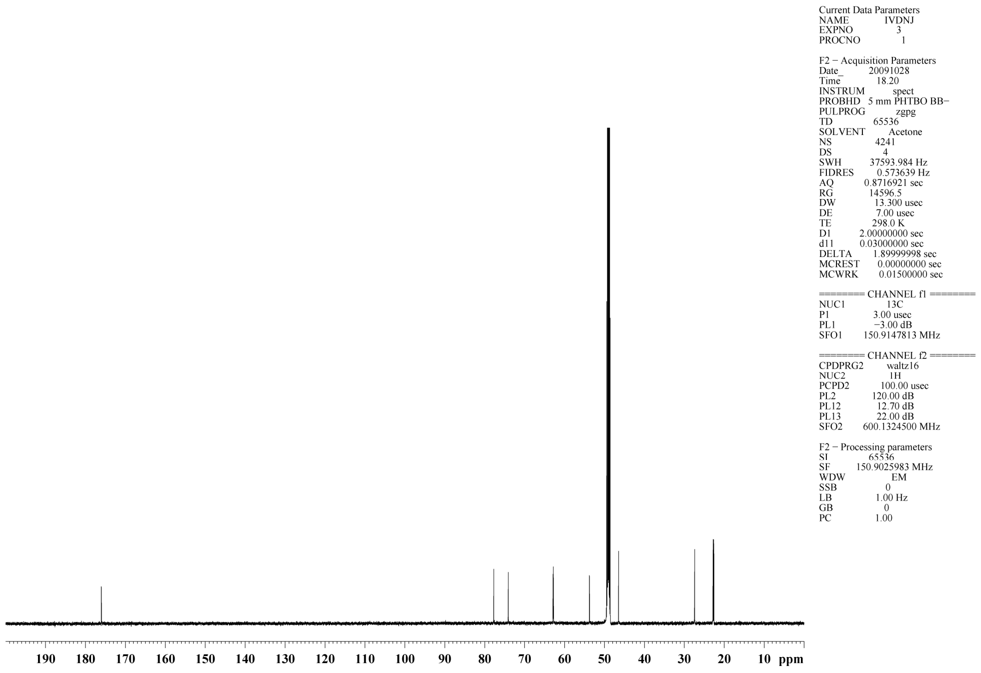


$^{13}$C NMR of **25**.
